# Study of 1,3-Dipolar Cycloaddition Between 4-Acyl-1H-pyrrole-2,3-diones Fused at the [e]-Side with a Heterocyclic Moiety and Diphenylnitrone: A Comprehensive MEDT, Docking Approach and MD Simulation

**DOI:** 10.3390/molecules30183718

**Published:** 2025-09-12

**Authors:** Soukaina Ameur, Agnieszka Kącka-Zych, Ziad Moussa, Reem I. Alsantali, Abdellah Zeroual, Mustafa S. Alluhaibi, Abdulrahman A. Alsimaree, Saleh A. Ahmed

**Affiliations:** 1 Molecular Modelling and Spectroscopy Research Team, Faculty of Science, Chouaïb Doukkali University, P.O. Box 20, El Jadida 24000, Morocco; ameursoukaina8@gmail.com (S.A.); r.alsantli@tu.edu.sa (A.Z.); 2 Cracow University of Technology, Faculty of Chemical Engineering and Technology, Department of Organic Chemistry and Technology, Warszawska 24, 31-155 Cracow, Poland; 3 Department of Chemistry, College of Science, United Arab Emirates University, Al Ain P.O. Box 15551, United Arab Emirates; 4 Department of Pharmaceutical Chemistry, College of Pharmacy, Taif University, P.O. Box 11099, Taif 21944, Saudi Arabia; zeroualabdellah2@gmail.com; 5 Department of Chemistry, Faculty of Science, Umm Al-Qura University, Makkah 21955, Saudi Arabia; msluhaibi@uqu.edu.sa; 6 Department of Chemistry, College of Science and Humanities, Shaqra University, Shaqra 11911, Saudi Arabia; alsimaree@su.edu.sa

**Keywords:** 1,3-dipolar cycloaddition, 1H-pyrrole-2,3-diones, nitrones, density functional theory, molecular docking, MD simulation

## Abstract

In this article, the 1,3-dipolar cycloaddition (1,3-DC) reactions between 4-acyl-1H-pyrrole-2,3-diones fused at the [e]-side with a heterocyclic moiety (FPDs) and diphenylnitrone are studied using Molecular Electron Density Theory (MEDT) at different computational levels. An analysis of the global reactivity descriptors has determined the role of the reagents. FPDs will act as electrophiles, while diphenylnitrone will be a nucleophile. It was found that the reactions proceed according to a one-step but asynchronous mechanism. Additionally, based on the Bonding Evolution Theory (BET) analysis of the model 1,3-DC reaction between FPDs **1b** and diphenylnitrone **2**, we can distinguish eight different phases. The formation of the first C1-O5 single bond takes place in phase VII through the disappearance of the V(C1) monosynaptic basin and the depopulation of the V″(O5) monosynaptic basin, while the formation of the second C2-C3 single bond begins at the last phase of the reaction through the connection of two V(C2) and V(C3) monosynaptic basins. Based on this, we can classify this reaction as a “*one-step two-stage*” process. Furthermore, molecular dynamics (MD) simulation analysis up to 100 ns demonstrated the stability of both the 2P3B–Ligand1 and 2P3B–Zidovudine complexes. An enhancer of shape compression was generated for ligand1, whereas Zidovudine generated a more packed and stable hydrogen bond network that would allow a better occupancy of the active site.

## 1. Introduction

**Figure 1:**
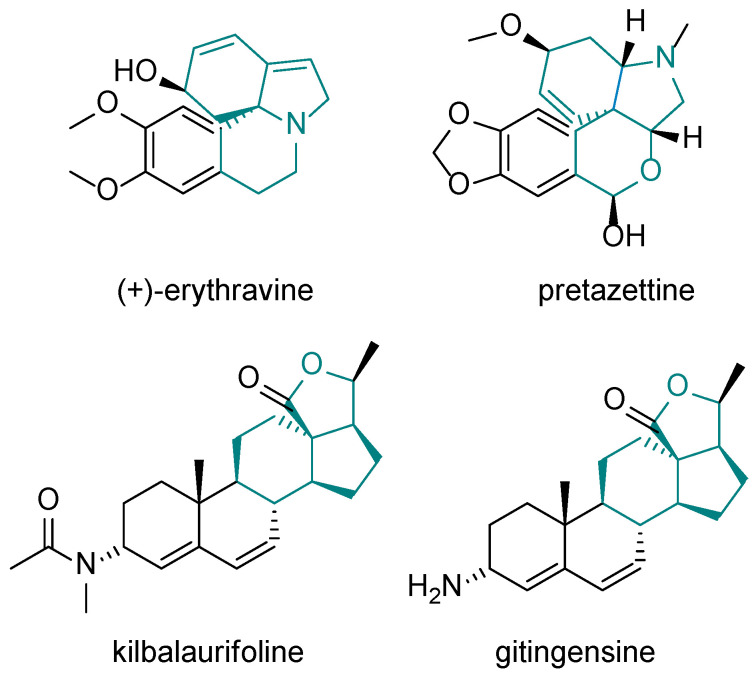
Examples of biologically active natural products bearing a polycyclic spirotypic motive.

Currently, attention in drug discovery is focused on small molecules with complex 3D structures. Such molecules are finding applications in molecular biology and medicine by reducing promiscuous binding and increasing the chance of drug delivery [1]. From this perspective, the spiro polycyclic framework (Figure 1) is a promising model for the design of complex 3D scaffolds. Moreover, this scaffold is a central component of many biologically active natural products [2].

**Scheme 1:**
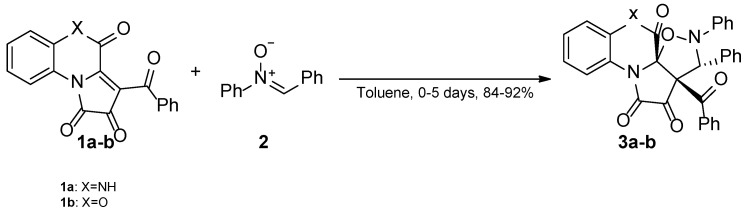
Experimentally observed path of the 1,3-dipolar cycloaddition reaction between FPDs (**1a-b**) and diphenylnitrone (**2**).

The 1,3-dipolar cycloaddition (1,3-DC) reaction has been shown to be a simple tool for simultaneously introducing several stereocenters in a single step to obtain complex 3D structures [3]. These syntheses have contributed to the preparation of numerous spiro polycyclic compounds [4]. Furthermore, 1H-pyrrole-2,3-diones exhibit polyelectrophilic properties, making them capable of synthesizing heterocyclic moieties with diverse skeletons [5]. On this basis, the synthesis of several 3D complex spiro polyheterocycles has been developed. Stepanova [6] hypothesized that 4-acyl-1H-pyrrole-2,3-diones fused at the [e]-side with a heterocyclic moiety (FPDs) (**1a-b**) could provide access to specific 6/6/5/5-tetracyclic systems (**3a-b**), cytotoxic alkaloids, kibalaurifoline, and gitingensin, via a 1,3-DC reaction with diphenylnitrone (**2**) (Scheme 1).

The 1,3-dipolar cycloaddition, in particular ([3+2] cycloaddition reaction (32CA)) reactions of nitrones with different types of dipolarophiles, has been investigated by both experimental and computational methodologies. Over the past few years, Density Functional Theory (DFT) and Molecular Electron Density Theory (MEDT) inspired studies have characterized the concerted but solvent asynchronous nature of these reactions, the Bonding Evolution Theory (BET), and the factors controlling regio- and stereo-selectivity [7,8,9,10,11]. Exploiting this model, case studies on nitronemaleimide, N-t-butyl nitronecyanoacetylene, and N-t-butyl nitronenitroethene have been conducted to reveal the way in which the polarity of 32CA (controlled by strong acceptors on the dipolarophile) diminishes the barriers and shapes the reaction trajectory [7,8,9,10]. Furthermore, conceptual DFT descriptors (electrophilicity/nucleophilicity indices, Parr functions) as well as Global Electron Density Theory (GEDT) metrics justify the selectivity in regio- and endo/exo direction in similar nitrone systems [7,11]. Together, these DFT/MEDT guidelines provide a predictive framework for designing nitrone 32CA with an aimed site selectivity and useful efficiency, therefore bridging between theoretical predictions and experimentally observed product ratios [7,8,9,10,11].

**Scheme 2:**
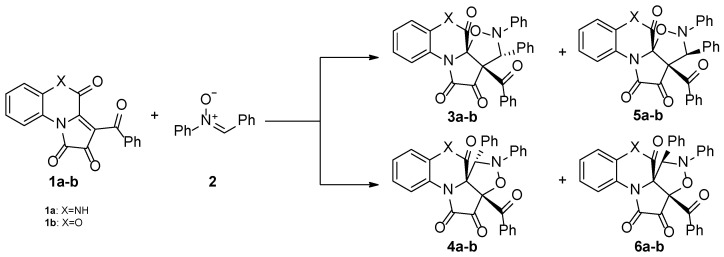
Theoretically possible path of the 1,3-DC reaction between FPDs (**1a-b**) and diphenylnitrone (**2**) leading to products **3a-b**, **4a-b**, **5a-b**, and **6a-b**.

In the conducted studies, the authors [6] did not pay attention to the fact that it is theoretically possible to obtain four regioisomeric (**3a-b** and **4a-b**) and stereoisomeric (**5a-b** and **6a-b**) products (Scheme 2). The experimental studies conducted also do not include a detailed understanding of the reaction mechanism. Therefore, we decided to fill this gap by performing studies on the basis of the Molecular Electron Density Theory (MEDT) [12]. Recently, MEDT analysis has been very successful in revealing the mechanisms of many reactions [13,14,15,16,17,18]. In the present work we performed the study of the electronic structure of the reactants, analyzed the reaction profiles, and proposed the accurate reaction mechanism. In addition, the products under study was subjected to the ADMET survey, molecular dynamics simulation, and in-depth molecular docking studies against the HIV-1 target protein. The binding modes and affinities of the interactions were calculated to determine the compounds with inhibitory potential, suggesting that they could be used as candidates for lead development in antiviral drugs.

## 2. Results and Discussion

The research results are discussed in four different parts: (i) in Section 2.1, an Electron Localization Function (ELF) topological analysis and a Natural Population Analysis (NPA) of the reactants are carried out in order to characterize their electronic structure; (ii) in Section 2.2, the reaction profiles associated with the 1,3-DC between FPDs (**1a-b**) and diphenylnitrone (**2**) are analyzed; (iii) in Section 2.3, the Bonding Evolution Theory study of the favorable path of reaction between FPD (1b) and diphenylnitrone (2) is analyzed for establishing the molecular mechanism; (iv) and finally, in Section 2.4, molecular dynamics (MD) simulations are performed.

### 2.1. Analysis of the Electronic Structure of the Reagents

**Figure 2:**
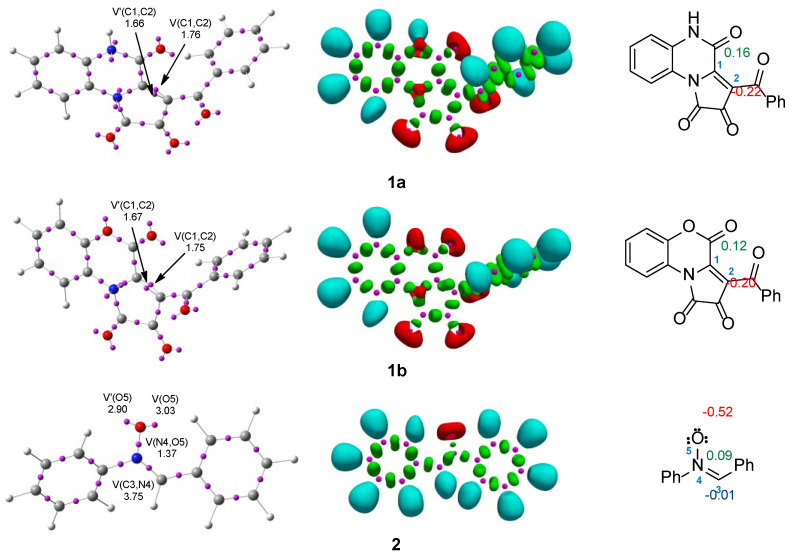
ELF localization domains and basin attractor positions together with the most significant valence basin populations and proposed Lewis-like structures of FPDs (**1a-b**) and diphenylnitrone (**2**).

At the beginning we decided to analyze the electronic structure and reactivity indices of the reagents (**1a-b**, **2**). Therefore, using the ELF analysis [19] together with the NPA study [20,21], we proposed Lewis-like structures with ELF localization domains and the most important valence basin populations for FPDs (**1a-b**) and diphenylnitron (**2**), which are presented in Figure 2.

The ELF picture of the FPDs (**1a-b**) in the most important region shows the two V (C1,C2) and V′(C1,C2) disynaptic basins (integrating in **1a**: 1.66e and 1.76e; and in **1b**: 1.67e and 1.75e, respectively) (Figure 2). These disynaptic basins are associated with the presence of a double bond between the C1 and C2 carbon atoms. In turn, in the ELF structure of diphenylnitrone (**2**), we can distinguish one V(C3,N4) disynaptic basin, integrating 3.75e, related to the double bond between the C3 and N4 atoms. In the most important region of **2**, we also observe the presence of one V(N4,O5) disynaptic and two V(O5) and V′(O5) monosynaptic basins associated with one N4-O5 single bond and free electron pairs on the oxygen atom (Figure 2).

The NPA of FPDs (**1a-b**) indicates that the C1 carbon atom in both molecules has a positive charge, 0.12e and 0.16e, respectively, while the other C2 carbon atom has a negative charge, −0.20 in **1a** and −0.22 in **1b**, which form the double bond. For molecule **2**, the natural atomic charge for the O5 atom is −0.52e (Figure 2). In turn, the natural atomic charge for the N4 atom is 0.09, while the C3 atom has a neutral charge.

Next, we also analyzed the role of reagents in the 1,3-DC reaction based on analysis of the global indices, defined within the context of the conceptual density functional theory (CDFT) [22,23], which are collected in Table 1. The electronic potential [24] µ of **1a** and **1b** (4.85 eV for **1a** and 5.14 eV for **1b**) is higher than that for the second reactant **2** (µ = 3.57 eV). Based on the analysis of the chemical potential of the reactants, we can conclude that GEDT [25] in these 1,3-DC reactions will proceed from diphenylnitrone (**2**) towards FPDs (**1a-b**). The calculated index of electrophilicity [26,27] ω of the FPDs (**1a-b**) is 4.13 eV and 4.63 eV, respectively, On the other hand, the calculated index of nucleophilicity [22] N for these compounds is 2.85 eV and 2.55 eV, respectively. On the basis of this analysis, we can classify these compounds as strong electrophiles and moderate nucleophiles [21]. In turn, the analysis of global reactivity descriptors of diphenylnitrone (**2**) indicated that index of electrophilicity [22] ω of **2** and index of nucleophilicity [22] N are 1.67 eV and 3.64 eV, respectively. According to that, we can state that **2** is classified as a strong electrophile and strong nucleophile [21]. Based on the performed calculations, we have determined the role of the reagents in the analyzed 1,3-DC reactions. Compounds **1a,b** will act as electrophiles, while compound **2** will be a nucleophile.

According to the electrophilic P_k_^+^ Parr functions [26] of FPDs (**1a-b**) and the nucleophilic P_k_^−^ Parr functions [26] of **2** (Table 2), we determined the most nucleophilic and electrophilic centers in reagents. These data are presented in Table 2. Analysis of the electrophilic P_k_^+^ Parr functions of FPDs (**1a-b**) indicates that the most electrophilic center is located on the C1 carbon atom (**1a**: P_kC1_^+^ = 0.27 eV; **1b**: P_kC1_^+^ = 0.25 eV). In turn, analysis of nucleophilic P_k_^−^ Parr functions of **2** indicates that the most nucleophilic center is located on the O5 oxygen atom (**2**: P_KO5_^−^ = 0.42 eV). It is worth noting that the C3 carbon atom shows a much lower value of nucleophilic P_k_^−^ Parr functions (**2**: P_kC3_^−^ = 0.16 eV). Based on the electrophilic P_k_^+^ and nucleophilic P_k_^−^ Parr functions, we can state that the 1,3-DC reaction between FPDs (**1a-b**) and diphenylnitrone (**2**) is realized through interactions of the C1 carbon atom for **1a-b** with the O5 oxygen atom of **2**. It is worth emphasizing here that the performed calculations are in full agreement with the experimental results [23].

### 2.2. The Reaction Profiles Associated with the 1,3-DC Between FPDs (***1a-b***) and Diphenylnitrone (***2***)

It was found that all reactions proceed according to a one-step mechanism. Only one transition state (TS) was localized in the analyzed reaction pathway. The pre-reaction complex (MC) is formed first. This is accompanied by a reduction in the Gibbs free energy of the reaction path to −3.7 kcal/mol for **MC1a** and −3.9 kcal/mol for **MC2a** according to the B2PLYP-D3/ma-def2-TZVPP calculations (Table 3).

**Figure 3:**
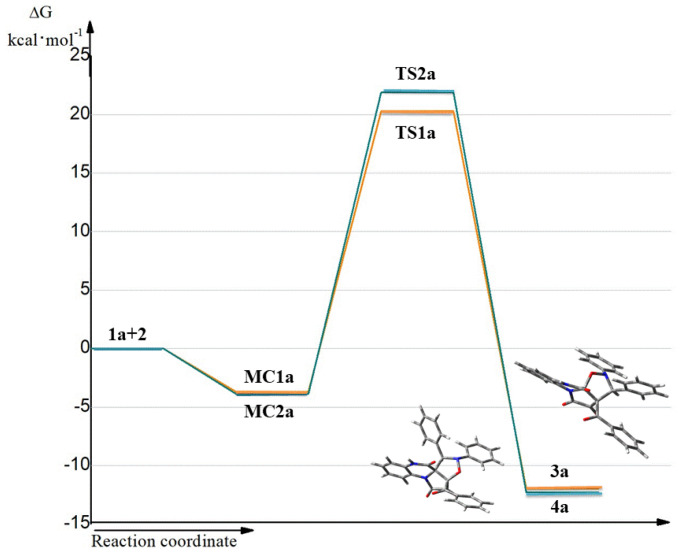
Gibbs free energy profile for the 1,3-DC reaction between FPD (**1a**) and diphenylnitrone (**2**) in the light of B2PLYP-D3/ma-def2-TZVPP calculations.

**Figure 4:**
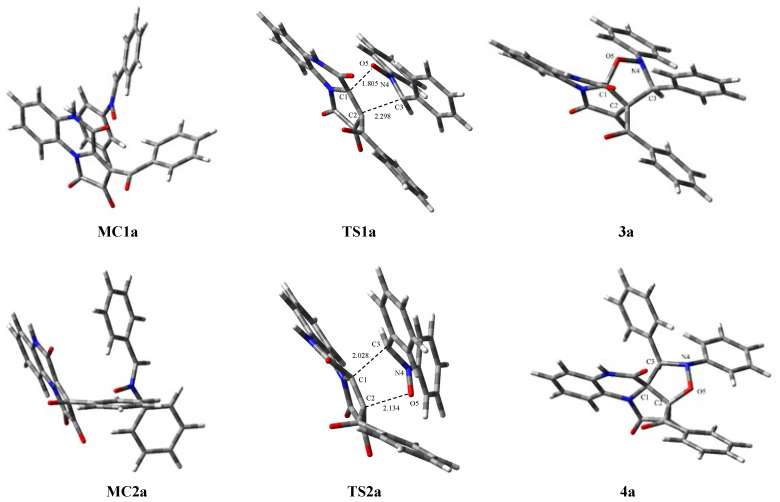
Views of stationary structures of processes in the 1,3-DC reaction between FPD (**1a**) and diphenylnitrone (**2**) in the light of B2PLYP-D3/ma-def2-TZVPP calculations. Distances are given in angstroms (Å).

Further transformation of the reaction system proceeds into the transition state (TS). The formation of the TS is associated with an increase in the Gibbs free energy to 20.3 kcal/mol for **TS1a** (Figure 3). It is worth paying attention to the fact that the Gibbs free energy for **TS2a** is 21.9 kcal/mol. The reaction leading to product **4a** is also kinetically allowed. Within the TSs, we observe the formation of two single bonds (Figure 4). Based on the TSs, we can conclude that these bonds are not formed at the same time. As a result, TS structures can be considered as asynchronous. The further reaction path leads to the products.

Similar values of Gibbs free energy were obtained using the M06-2X/6-311+G(d,p) computational level (Table S2 in Supplementary Materials). The formation of TS1a is related to the increase in Gibbs free energy to the value of 20.5 kcal/mol, while for TS2a Gibbs free energy is 21.2 kcal/mol. The method using the aforementioned functional yields very similar energies to those obtained for the B2PLYP-D3/ma-def2-TZVPP calculation and also indicates the asynchronous character of the reaction.

We also analyzed theoretically possible reaction pathways leading to stereoisomeric products **5a-b** and **6a-b** (Scheme 2). However, the formation of the transition states (**TS3a-b**, **TS4a-b**) leading to these products requires significant Gibbs free energy (Table S1). Therefore, the formation of these products is forbidden from a kinetic point of view, and the results of quantum chemical calculations fully confirm the experimental results [6]. Quantum-chemical calculations confirm the diastereoselectivity of the analyzed reactions and exclude the theoretically possible pathway leading to products **5a-b** and **6a**-**b**.

We also performed calculations using various functionals and basis sets. Thermodynamic parameters for 1,3-DC reactions of FPDs (**1a-b**) and diphenylnitrone (**2**) conducted using various functionals and basic sets are located in Supplementary Materials in Tables S1–S4. Calculations using other levels of theory also indicate a one-step mechanism for the analyzed reactions. It is worth noting, that the calculations indicate lower Gibbs free energy barriers, which indicates a more straightforward course of the analyzed reaction. It should be noted that all analyzed theoretical levels fully confirm the reaction proceeding according to the asynchronous mechanism, despite significant differences in energy values.

The structure of the **TS1a** is a typical structure for cycloaddition reactions. The O5-C1 bond lengths for all TSs located in the **1a+2** reaction are much smaller than C2-C3. This observation is in agreement with the most favorable interactions between the most nucleophilic and electrophilic centers. Analysis of the structure of the TSs involved in the reaction of FPD (**1a**) and diphenylnitrone (**2**), leading to two products: **3a** and **4a**, indicates that they are strongly asynchronous (Figure 4).

### 2.3. A BET Study of the 1,3-DC Reaction Between FPDs (***1b***) and Diphenylnitrone (***2***)

**Figure 5:**
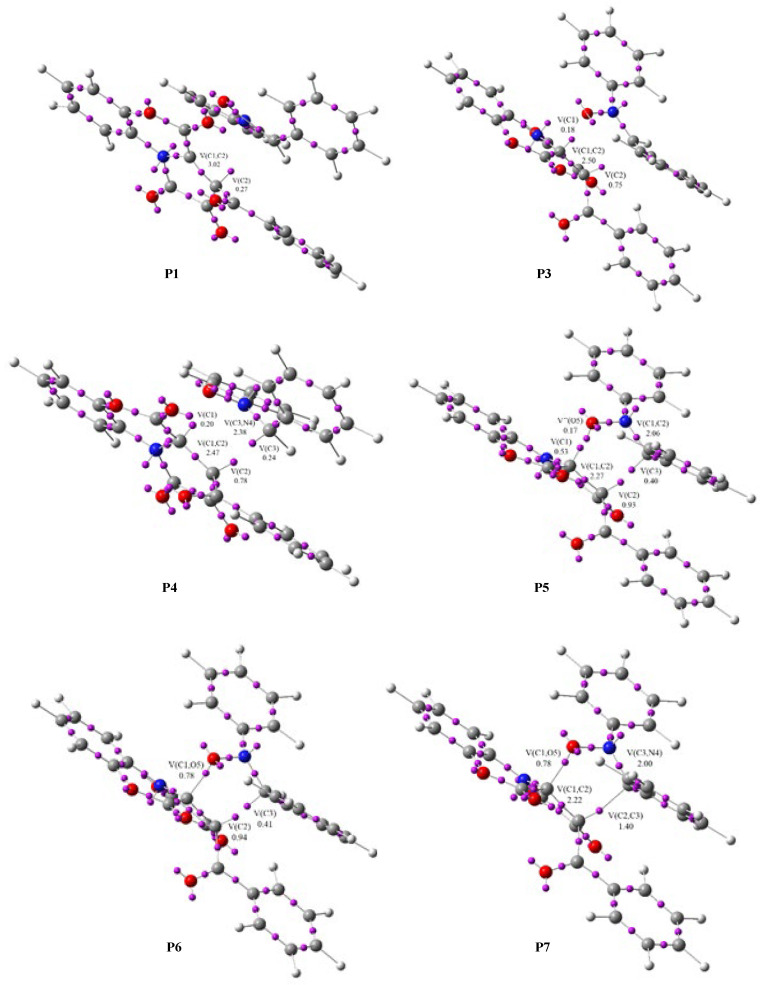
The most significant attractor positions of the ELF valence basins of the structures **P1**, **P3–P7** participating in the C2-C3 and C1-O5 single bond formation participating in the 1,3-DC reaction between FPDs (**1b**) and diphenylnitrone (**2**). The electron populations, in average number of electrons, are given in e.

This quantum-chemical analysis will allow for understanding the changes occurring during the reaction, the nature of the reaction, and the establishment of the mechanism of the reactions. The results of the BET analysis are presented in Table 4 and Figure 5.

*Phase I*, 3.06 Å ≥ d(C2-C3) > 2.90 Å and 2.59 Å ≥ d(C1-O5) > 2.46 Å, begins at the molecular complex (**MC1b**), which is the first point where the reaction path between **1b** and **2** begins. In **P1** we observe only slight changes. Two V(C1,C2) and V′(C1,C2) disynaptic basins present in **1b** were merged into one new V(C1,C2) disynaptic basin in **MC1b**, integrating 3.28e (Table 4).

At **P1**, *Phase II* begins, 2.90 Å ≥ d(C2-C3) > 2.47 Å and 2.46 Å ≥ d(C1-O5) > 2.05 Å. At this point, the V(C2) monosynaptic basin is created, integrating 0.27e, by the depopulation of the V(C1,C2) disynaptic basin. This change is related to the formation of the *pseudoradical* center at the C1 carbon atom (Figure 5).

In *Phase III*, 2.47 Å ≥ d(C2-C3) > 2.32 Å and 2.05 Å ≥ d(C1-O5) > 1.90 Å, which begins at **P2**, we observed the formation of a new V(N4) monosynaptic basin, integrating 0.82e, by depopulation of the V(C3,N4) disynaptic basin. This change is related to the creation of a lone pair of electrons in the N4 nitrogen atom (Table 4).

**Figure 6:**
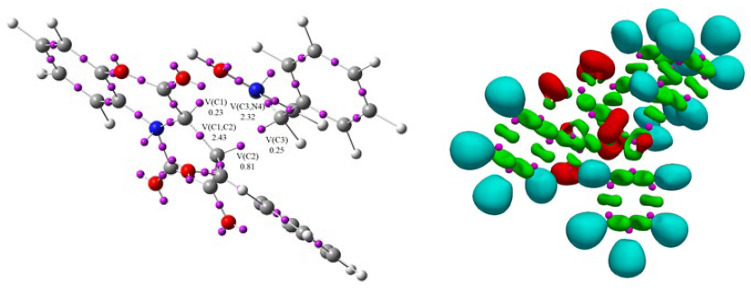
The most significant attractor positions of the ELF valence basins of the structure **TS1b** participating in the C2-C3 and C1-O5 single bond formation participating in the + 1,3-DC reaction between FPDs (**1b**) and diphenylnitrone (**2**). The electron populations, in average number of electrons, are given in e.

In the next two phases, *Phases IV* and *V,* (*Phase IV*: 2.47 Å ≥ d(C2-C3) > 2.32 Å and 2.05 Å ≥ d(C1-O5) > 1.90 Å; *Phase V*: 2.29 Å ≥ d(C2-C3) > 2.11 Å and 1.87 Å ≥ d(C1-O5) > 1.71Å), which start in **P3** and **P4**, we observed the next most relevant change along the IRC. These phases are related to the formation of two new monosynaptic basins, V(C1) and V(C3), integrating 0.18e and 0.24e, respectively (Figure 5 and Table 4). These changes are related to the formation of two *pseudoradical* centers on C1 and C3 atoms. The transition state of **TS1b** of this reaction is found in this phase, d(C2-C3) = 2.268 Å and d(C1-O5) = 1.850 Å (Figure 6).

In *Phase VI*, 2.29 Å ≥ d(C2-C3) > 2.10 Å and 1.71 Å ≥ d(C1-O5) > 1.70 Å, which begins at **P5**, the next significant topological change along the reaction takes place. At this point we see the creation of a new V″(O5) monosynaptic basin, with an initial population of 0.17e (see **P5** in Figure 5). This monosynaptic basin was created by dividing the population of the two V(O5) and V′(O5) monosynaptic basins (Table 4).

*Phase VII*, 2.10 Å ≥ d(C2-C3) > 2.06 Å and 1.70 Å ≥ d(C1-O5) > 1.67 Å, begins at **P6**. In this phase, we observed the formation of a new V(C1,O5) disynaptic basin, integrating 0.78e, together with the disappearance of the V(C1) monosynaptic basin and the depopulation of the V″(O5) monosynaptic basin (Table 4). This change is associated with the formation of the first C1-O5 single bond.

Finally, the last phase, *Phase VIII*, 2.06 Å ≥ d(C2-C3) > 1.76 Å and 1.67 Å ≥ d(C1-O5) > 1.49 Å, is located between points **P7** and **3b**. Here, a new V(C2,C3) disynaptic basin integrating 1.40e is observed together with the disappearance of two V(C2) and V(C3) monosynaptic basins. These changes are related to the formation of a new C2-C3 single bond present in **3b**.

**Scheme 3:**
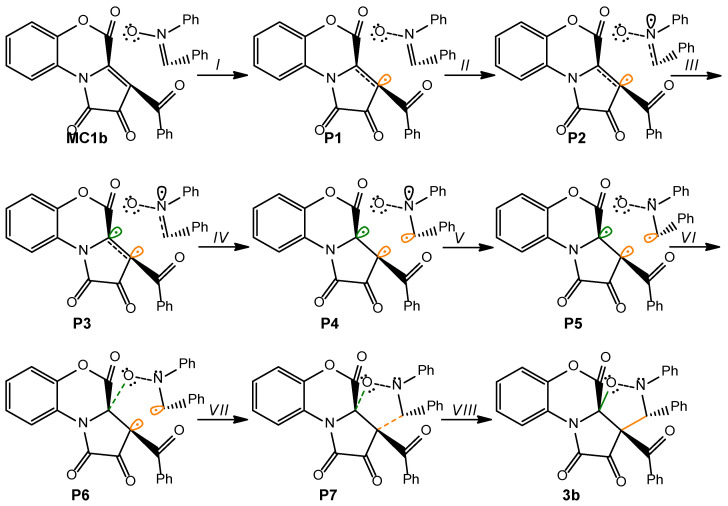
The molecular mechanism of the in 1,3-DC reaction between FPDs (**1b**) and diphenylnitrone (**2**) by Lewis-like structures resulting from the topological analysis of the ELF along the reaction path.

Based on the BET study, we can draw the following conclusions regarding the model 1,3-DC reaction between FPDs (**1b**) and diphenylnitrone (**2**). (i) This 1,3-DC reaction takes place through eight different phases (Scheme 3); (ii) the formation of the first C1-O5 single bond takes place in *Phase VII* through the disappearance of the V(C1) monosynaptic basin and the depopulation of the V″(O5) monosynaptic basin; (iii) the formation of the second C2-C3 single bond begins in the last phase of the reaction through the connection of two V(C2) and V(C3) monosynaptic basins; (iv) based on the BET analysis, we can classify this reaction as a “one-step two-stage” process.

### 2.4. Molecular Docking Study

**Figure 7:**
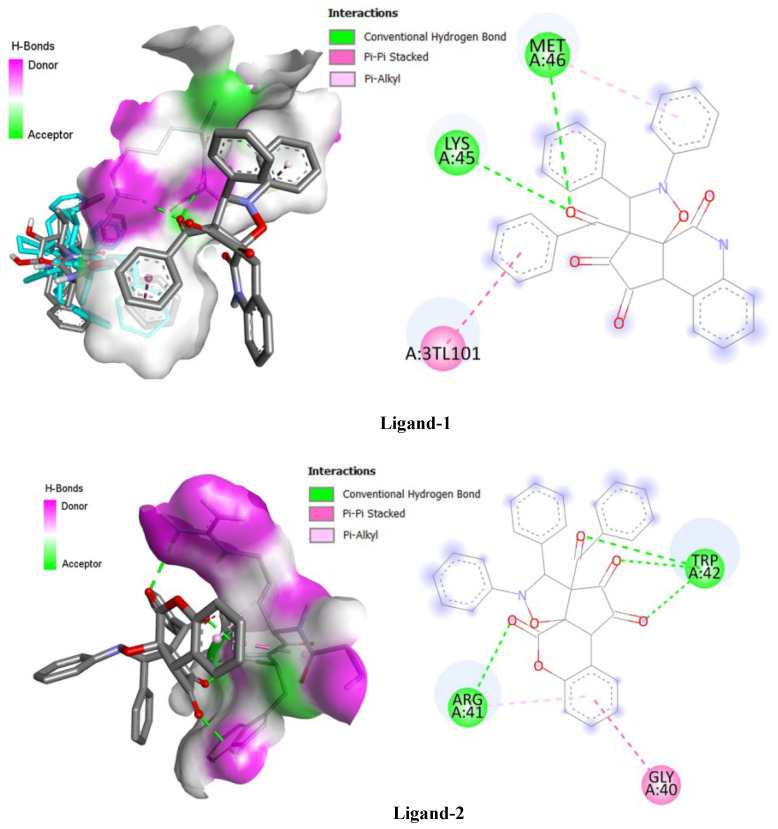
Two-dimensional and three-dimensional interactions of the studied elements with the ten residual active sites of the 2P3B receptor protein.

Molecular docking is a common computational method widely used in medicine and biology; most of the time it aims to discover the binding pattern of a ligand with its target protein [24]. Not only does it allow us to estimate the binding affinity but also provides crucial data to design and optimize novel pharmacologically active chemicals by modeling and predicting molecular interaction modes. Molecular docking is one of the most useful tools, which is used to establish the high-resolution binding conformation and pose of a ligand in an active site on the target protein that might accelerate the drug discovery process. Table 5 shows a summary of the affinity energies obtained for the family of compounds studied, and Figure 7 demonstrates two- and three-dimensional interactions formed with active site residues in receptor protein (PDB code: 2P3B) by these ligands. Docking protocol was conducted using AutoDock Vina (version 2021). The crystallographic structure of protein 2P3B was obtained from the PDB database (PDB ID: 2P3B), and all the structures were converted into PDBQT format using AutoDockTools software (version 2021). The three-dimensional calculation box was defined by sides of length 100 × 100 × 100 Å centered at the coordinates (x = −25.995, y = 12.591, z = 59.151). Three-dimensional visualizations of the complexes produced were performed using PyMOL and Discovery Studio software, thus enabling an accurate and meaningful interpretation of results at a structural level.

Figure 7 illustrates the molecular interactions established between two tested ligands (Ligand-1 and Ligand-2) and the active site of the target protein 2P3B, through three-dimensional and two-dimensional representations. These visualizations allow for a better understanding of the nature of the non-covalent interactions that stabilize the ligand-receptor complex and for the interpretation of the calculated binding affinities presented in Table 5.

In 3D, the formed complexes show specific interactions involving conventional hydrogen bonds (in green), π–π stacked interactions (in pink), and hydrophobic π–alkyl type interactions (in purple). These interactions are located at the active site of the protein, whose molecular surface is represented in transparency to visualize the insertion of the ligand into the catalytic cavity. On the two-dimensional level, the interactions between Ligand-1 and the protein notably involve residues MET A:46, LYS A:45, and A:3TLU101, suggesting a strong and well-oriented interaction. On the other hand, Ligand-2 primarily interacts with residues TRP A:42, ARG A:41, and GLY A:40, with a reduced number of hydrogen bonds.

These observations are consistent with the binding affinities obtained by docking (Table 5). Indeed, Ligand-1 exhibits an energy affinity of −6.9 kcal/mol, higher than that of Ligand-2 (−5.0 kcal/mol) and the reference compound AZT (−3.9 kcal/mol). This better affinity can be attributed to the multiplicity and nature of the stabilizing interactions formed by Ligand-1 within the active site. Thus, this comparative analysis highlights the higher inhibitory potential of Ligand-1, reinforcing its interest as a potential pharmacological candidate targeting the 2P3B protein.

### 2.5. Simulation of Molecular Dynamics

Molecular dynamics, a sophisticated computerized simulation technique, is used to evaluate the microscopic stability of proteins and protein-ligand complexes. It is shown that structure, function, fluctuation, interaction, and behavior accomplish this. GROMACS version 2023.1 was utilized for MD computations to examine the behavior of the two complexes. General Force CHARMM protein content is field parameterized. Ligand topologies were implemented by the SwissParam server. Steepest descent was used to vacuum minimize the structures 2500 times to address steric concerns. The structure was solvated by the SPC water model. The system was balanced using the gmx genion instrument after the addition of Na+ and Cl- ions. This was performed to guarantee the electrical neutrality of the system. MD simulations entered production, NVT, and NPT after minimization. The systems were balanced in two phases. Initially, particle number, volume, and temperature were maintained by performing a 100 picosecond NVT equilibration. Raising the system temperature to 300 kelvin was the goal of the process. To attain temperature, pressure, and particle number homogeneity, the second stage required an exact 100 picosecond NPT equilibration. Maintaining system pressure and density was crucial. During simulations, bond restrictions on all bonds restricted the placement of the protein group. Because NVT and NPT relaxed the protein by limiting water molecules around it, the system entropy decreased. Molecular dynamics using the v-rescale thermostat and the Parrinello-Rahman barostat approach. For 100 picoseconds, the barostat and thermostat were set. Covalent bonding was constrained using the Linear Constraint Solver program (version 2023). Chemical bond interactions were handled using the sophisticated Particle-Mesh Ewald (PME) approach. Every system has a 100 ns production run after equilibrium.

#### 2.5.1. Root Mean Square Deviation (RMSD)

RMSD is an expression of the difference between two frames. It is calculated for each frame from the profile. The root mean square deviation of frame x is computed.

$${RMSD}_{x}=\sqrt{\frac{1}{N}\sum_{l=1}^{N}\left(r_{i}^{\prime }\left(t_{x}\right)\right)-{r_{i}\left(t_{ref}\right)}^{2}}$$

**Figure 8:**
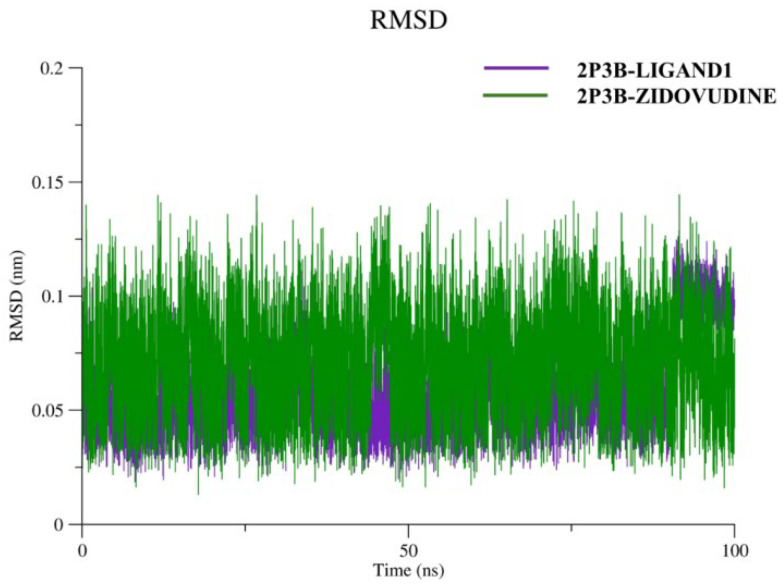
Comparative analysis of the structural stability of the 2P3B–Ligand1 and 2P3B–Zidovudine complexes via RMSD.

The set that is picked has N atoms all together. The reference time, t_ref_, is usually the first frame, t = 0. The positions of the selected atoms in frame x, after alignment to the reference frame, are referred to as r′. Frame x corresponds to capture time t_x_ All frames of computational trajectories are processed by experts [28]. The MD trajectory parameter file was visualized with XmGrace (version 2013) [29]. RMSD is given in Figure 8.

The RMSD plot demonstrates the structural stability of the 2P3B–Ligand1 (purple) and 2P3B–Zidovudine (green) complexes throughout 100 ns of simulation. The overall conformational stability of both complexes is good, as indicated by the RMSD values (0.03–0.15 nm). Fluctuations of the complex of 2P3B–Zidovudine are mildly stronger but still acceptable, indicating the moderate flexibility of the complex in the active site. By contrast, in the 2P3B–Ligand1 complex, RMSD and RMSF were more stable and in order, reflecting a stable anchoring and a good, bound conformation. These findings are consistent with the fact that both ligands can effectively interact with the protein over the simulation, although a slight advantage in terms of stability and binding strength could be attributed to Ligand1.

#### 2.5.2. Root Mean Square Fluctuation (RMSF) Analysis for Atomic Flexibility

The Root Mean Square Fluctuation (RMSF) is an important factor in determining the local flexibilities of protein residues in molecular dynamics (MD) simulations. It is a measure of how much each residue is displaced from its average position during the simulation and can provide information on the dynamics of particular parts of the protein structure [30]. It was observed that the values of RMSF may be indicative of flexibile loops, stable secondary structures, or binding-induced rigidity in the macromolecule. In mathematical terms, the RMSF for a residue i is computed as:

$${RMSF}_{i}=\sqrt{\frac{1}{T}\sum_{t=1}^{T}\;{\left(r_{i}^{\prime }\left(t\right)-r_{i}\left(t_{ref}\right)\;\right)}^{2}}$$

**Figure 9:**
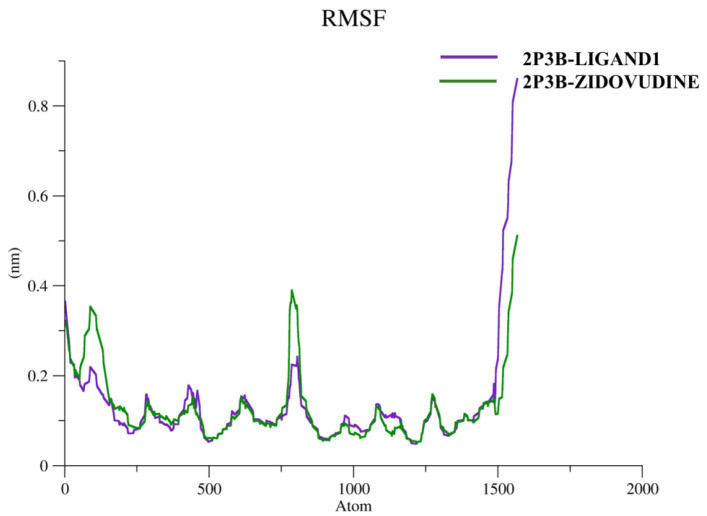
RMSF of 2P3B in complex with Ligand1 and Zidovudine during the simulation period.

RMSF is determined over trajectory time T. The reference time is t_ref_. The position of residue i is r_i_, and its atoms are r′ after superposition on the reference. The angle bracketed average square distance is calculated over residue atom selection. Protein regions with the highest simulated fluctuation are shown by the peaks on this map. N- and C-terminal protein tails vary more than other regions. RMSF figures are presented in Figure 9.

The flexibility of the individual residues within the 2P3B–Ligand1 and 2P3B–Zidovudine protein–ligand complexes during MD simulation was evaluated by the Root Mean Square Fluctuation (RMSF) analysis. As shown in the figure, the two complexes display low atomic fluctuations (RMSF < 0.4 nm) for most parts of the protein chain, being indicative of a stable and well-preserved structural core. It is interesting to report that the active sites region, which is the location where the inhibitors bind, exhibits very low fluctuation, which indicates that both Ligand1 and Zidovudine stay in the correct position during the simulation. But a significant enhancement of fluctuation can be found at the C-terminal part, especially of 2P3B–Ligand1; the peak is close to 0.85 nm. This increased mobility for the terminal loop could be indicative of increased solvent accessibility or lower packing density at this loop for the Ligand1 system. The nature of this tail flexibility does not affect the binding site in the binding region but may impact the overall conformational dynamics or allosteric behavior of the protein. In general, the RMSF profiles are consistent with the structural stability of the two complexes, indicating structural flexibility of the terminal residues.

#### 2.5.3. Radius of Gyration (Rg)

**Figure 10:**
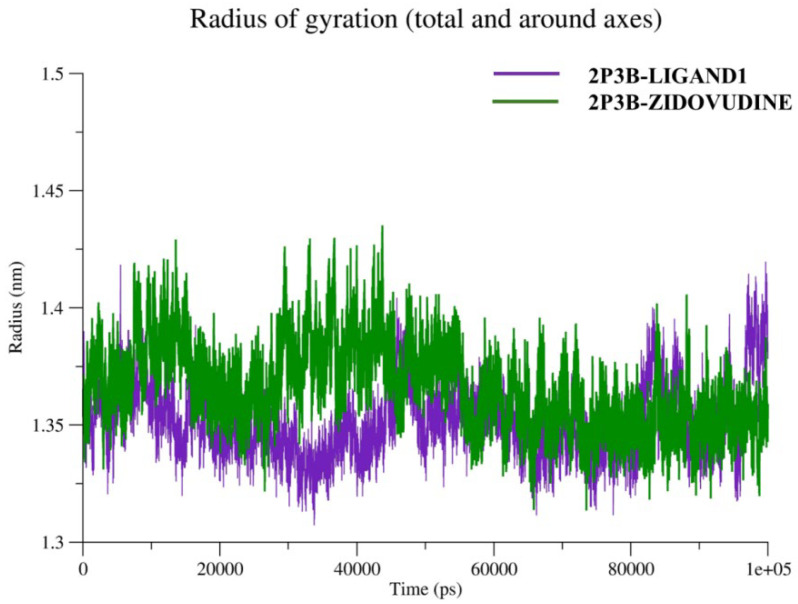
Radius of gyration profile of 2P3B bound to Ligand1 and Zidovudine up to 100 ns.

Radius of Gyration (Rg) is an important structural parameter representing the compactness and folding properties of a biomolecular system in molecular dynamics (MD) simulations. Rg is a mathematical property defined as the square root of the mean squared distance of all atoms (or a subset of atoms) from their center of mass (COM), allowing for mass weightage [31]. It is a measure of how closely or distantly the atoms are packed together in a molecule around its center. In protein-ligand complexes, the analysis of Rg during the simulation provides a criterion to assess the global structural stability. A constant Rg value over time would generally indicate that protein remains steadily compact, implying a lack of substantial unfolding/denaturation. On the other hand, observed variation in Rg can be interpreted as structural changes, denaturation, or conformational shifts in the molecule. In the present work, the Rg values were calculated for the 2P3B–Ligand1 and 2P3B–Zidovudine systems. This comparison clarifies whether the binding of each ligand affects the global compactness and conformational stability of the target protein. A stable and rigid low value of Rg throughout the simulation signifies a well-folded and stable protein–ligand complex needed for performing functional interactions in cellular systems. The radius of gyration for the 2P3B–Ligand1 and 2P3B–Zidovudine is given in Figure 10.

The analysis of radius of gyration (Rg) presented in the figure indicates that the 2P3B–ligand complexes retain compactness and overall structure stability globally during the simulation time. The 2P3B–Zidovudine complexes (green line) showed Rg distributions between 1.32 and 1.45 nm, with a mean value of 1.38 nm, indicating a slightly more relaxed but still relatively stable protein conformation. On the other hand, the Rg of the 2P3B–Ligand1 complex (violet line) was smaller and more stable and was generally hovering around 1.35–1.36 nm, suggesting that the structure was more compact and compacted. The similar pattern of Rg trajectory for both systems during 100 ns simulation time indicates that no large conformational changes or denaturation occurred. However, the overall insignificant shifts in average Rg suggest that Ligand1 can stabilize a more folded state of the protein in comparison with Zidovudine. This fact is consistent with the notion that the association of Ligand1 enhances the stability of the protein’s tertiary structure.

#### 2.5.4. Solvent Accessibility and Structural Compactness

**Figure 11:**
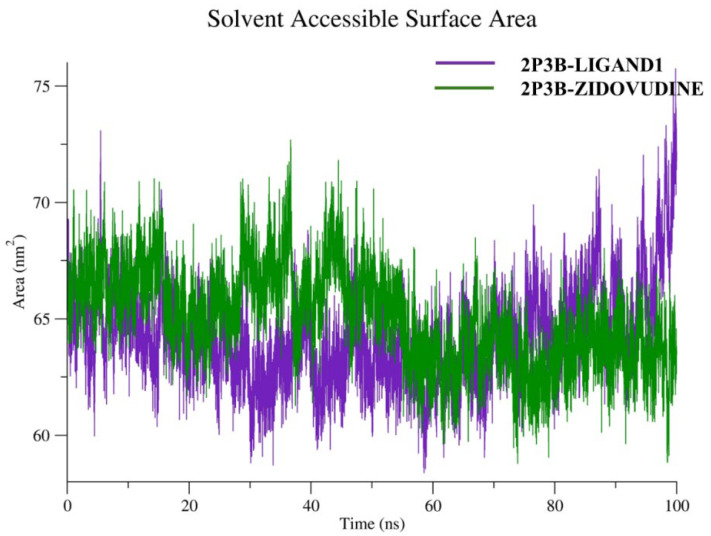
Solvent Accessible Surface Area (SASA) profiles of 2P3B–Ligand1 and 2P3B–Zidovudine complexes up to 100 ns.

The Solvent Accessible Surface Area (SASA) is an important criterion that describes the solvent-exposed surface of a biomolecule. It describes variations in molecular exposure and folding during molecular dynamics (MD) simulation. A steady plateau in the SASA value usually also means that the protein is keeping its one-to-one position with respect to the solvent during the simulation, while a significant fluctuation would imply a change in conformation or partial unfolding. SASA of the 2P3B–Zidovudine complex is given in Figure 11.

A Solvent Accessible Surface Area (SASA) analysis shows very different solvent exposure profiles within 100 ns molecular dynamics simulations for the two complexes. The 2P3B–Zidovudine complex (green curve) showed a corresponding SASA range of 63–75 nm^2^ (generally higher solvent exposure). The 2P3B–Ligand1 complex (violet curve), on the other hand, kept a little lower SASA value, in the range of 60–70 nm^2^, as average values. This lower surface exposure reflects a more contracted structural conformation and perhaps a better hydrophobic patch burial in the Ligand1-bound system. More importantly, the SASA was relatively steady over time in both complexes, suggesting that major conformational changes or unfolding did not occur and structural equilibrium was maintained during the simulations.

#### 2.5.5. Ligand-Receptor Hydrogen Bonding Studies

**Figure 12:**
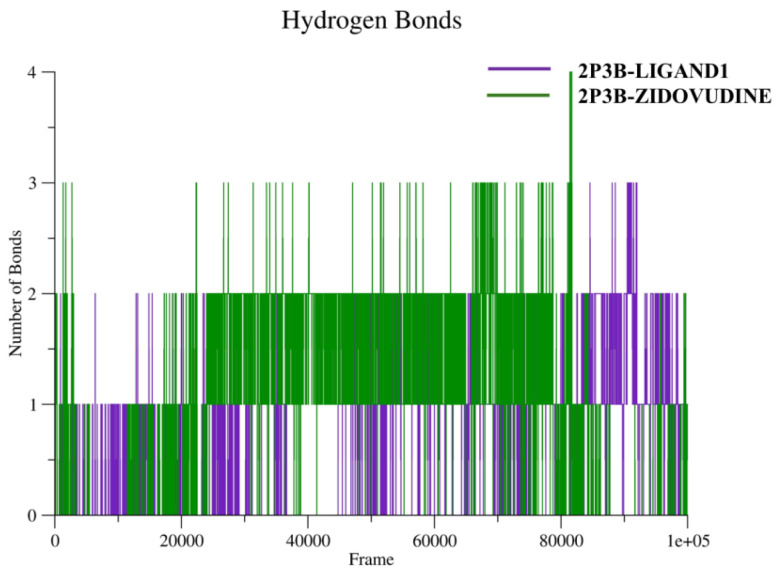
Temporal variation of hydrogen bonds between 2P3B and each ligand during the MD simulations.

During MD simulations, H-bonds are important for stabilization of protein–ligand complexes. Plotting the number of hydrogen bonds throughout a simulation reveals dynamics of both the number and strength of hydrogen bonds formed with ligand over time. Normally hydrogen bonds are persistent and have favorable binding energies, and thus the stable hydrogen bond profile contributes to the stability of the complex during the simulation. The hydrogen bonds of the complexes are shown in Figure 12.

The hydrogen bond profile shows different interaction behaviors of the two complexes. The 2P3B–Zidovudine (green) complex consistently adopted between 1 and 3 hydrogen bonds at all timescales, with some temporary spikes up to 4. This unaltered hydrogen-bonding arrangement could indicate strong and stable intermolecular interaction, leading to an improved binding affinity and preservation of structure at the active site. In the 2P3B–Ligand1 complex (violet), a rather irregular pattern was observed with only 1 to 2 hydrogen bonds that fluctuated more. Ligand1 has less hydrogen bonding, and such an incomplete network may correspond to weaker or more transient interactions that may potentially contribute to differences in overall binding stability compared to Zidovudine.

#### 2.5.6. In Silico ADMET Profile of Compounds **3a** and **3b**

The ADMET (adsorption, distribution, metabolism, excretion, toxicity) profile is one of the crucial steps in drug discovery of potential drug targets. It permits a predictive assessment of the kinetics and safety of compounds before experimental confirmation. Through the prediction of characteristics (i.e., intestinal absorption, tissue distribution, hepatic metabolism, excretion pathways, and toxicological hazard), this in silico method allows the rational choice of the most interesting molecules. The ADMET profiles of 3a and 3b were compared to aid rationalizing structure investment and to optimize their therapeutic potential. All analyses were performed in the online host software Biosig (version 2008) [32]. The values of all the predicted parameters are summarized in Table 6.

The comparison ADMET study of these two compounds, **3a** and **3b**, indicates that there are several significant PK and TA disparities. As for absorption, the two items have good aqueous solubility (log S ≈ −3.6), indicating that their oral bioavailability may be moderate. This is, however, not the case of the intestinal permeability, as determined from the Caco-2 test: compound **3a** reveals a log Papp value of 1.01, indicative of high permeability, whereas **3b** has a negative value (−0.058), revealing low absorption. However, both drugs have an intestinal absorption rate of greater than 99%, which is still acceptable. They are, in general, characterized by an extremely low skin permeability (log Kp = −2.735), which is, on average, typical of substances not intended for transdermal delivery. Both are non-substrates of P-glycoprotein, and this enables their membrane passage; however, both could inhibit both forms of P-glycoprotein, resulting in drug–drug interactions if these are co-administered with other substrates.

In terms of distribution, the two compounds have low volumes of distribution (log VDss < −1.5), indicating that they are predominantly distributed into the plasma. The free fraction of compound 3a is greater (Fu = 0.246 vs. 0.16 for **3b**), suggesting a greater amount of unbound molecule for pharmacological action. The two compounds are blood–brain barrier impermeable (log BB < −0.5) and have very low permeability on the central nervous system level (log PS < −2), which decreases the possibilities of neurotoxic side effects.

The elimination of both compounds appears moderate, as indicated by low total clearance (log CL < 0), and neither of the two seems to be a substrate of the renal transporter OCT2, and presumably they are eliminated mainly by hepatic excretion, with minimal contribution from the kidney for the elimination of these compounds.

Finally, the toxicology data shows a generally good profile for both compounds, with minor discrepancies. Neither is mutagenic (negative AMES test) or a hERG type I inhibitor (low risk of proarrhythmia), but both are hERG II inhibitors, which may precipitate secondary unwanted cardiac effects. That is, the MTD is a little higher for **3a,** and chronic toxicity is the same. However, a key point is that **3b** would be predicted to be hepatotoxic, unlike the case of **3a**. Furthermore, the environmental toxin value of the derivative **3b** (log minnow toxicity = 2.88) is particularly high, which makes us cautious on ecotoxicological grounds.

## 3. Computational Methodology

The quantum-chemical calculations were performed using the B3LYP(PCM)/6-311G(d,p) level of theory with the Gaussian 16 package [33]. The PLGrid infrastructure (the “Ares” supercomputer) at the national computing center “Cyfronet”. The detailed methodology for the quantum-chemical calculations is provided in the Supplementary Information (see Supplementary Materials, page S4).

## 4. Conclusions

The 1,3-DC reactions between FPDs (**1a-b**) and diphenylnitrone (**2**) have been studied in detail using the B2PLYP-D3/ma-def2-TZVPP and different functional and basic sets.

An analysis of the role of reagents in the 1,3-DC reaction based on analysis of the global indices, defined within the context of the CDFT, was performed. Based on the analysis of the chemical potential of the reactants, we can conclude that GEDT in these 1,3-DC reactions will proceed from diphenylnitrone (**2**) towards FPDs (**1a-b**). Based on this analysis of global electrophilicity and nucleophilicity, we can classify reagents **1a-b** as strong electrophiles and moderate nucleophiles. In turn, the diphenylnitrone (**2**) is classified as a strong electrophile and strong nucleophile. According to that, **1a-b** will act as electrophiles, while compound **2** will be a nucleophile.

Reactions **1a+2** and **1b+2** proceed according to a one-step mechanism. The first reaction stage is the formation of an MC in both cases, which is related to the growth of the Gibbs free energy of the reaction path. Further transformation of the reaction system proceeds into the TS. The formation of the TS is associated with an increase in the Gibbs free energy to 20.3 kcal/mol for **TS1a** and 21.9 kcal/mol for **TS2a**. It is worth noting that the reaction leading to product **4a** is also kinetically allowed. Additionally, reactions using other functional and basic sets were analyzed. All calculations indicate a one-step mechanism of the analyzed reactions.

In order to understand the bonding changes along the **1b+2** reaction path, a BET analysis was performed. The analysis of the 1,3-DC reaction takes place through eight different phases. The formation of the first C1-O5 single bond takes place in *Phase VII* through the disappearance of the V(C1) monosynaptic basin and the depopulation of the V″(O5) monosynaptic basin. On the other hand, formation of the second C2-C3 single bond begins in the last phase of the reaction through the connection of two V(C2) and V(C3) monosynaptic basins. According to the BET analysis, we can classify this reaction as a “*one-step two-stage*” process.

In addition. Molecular dynamics of 100 ns demonstrated the reasonable stability of the 2P3B–Ligand1 and 2P3B–Zidovudine complexes. Ligand1 led to a more compacted structure, whereas Zidovudine formed a denser and stable network of hydrogen bonds and would favorably be more bound to the active site.

## Figures and Tables

**Table 1:** The global reactivity descriptors of FPDs (**1a-b**) and diphenylnitrone (**2**) calculated at the B3LYP/6-31G(d) level of theory. The electronic chemical potential μ, chemical hardness η, electrophilicity ω, and nucleophilicity N are reported for the studied reagents.

	µ [eV]	η [eV]	ω [eV]	N [eV]
**1a**	4.85	2.84	4.13	2.85
**1b**	5.14	2.85	4.63	2.55
**2**	3.57	3.82	1.67	3.64

**Table 2:** Local electronic properties of FPDs (**1a-b**) and diphenylnitrone (**2**); the electrophilic P_k_^+^ and nucleophilic P_k_^−^ and the indicies of local electrophilicity ω_k_ and nucleophilicity N_k_ are also included.

		$P_{k}^{+}$	ω_k_	$P_{k}^{-}$	N_k_
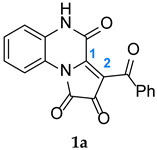	**C1**	0.27	1.11	−0.09	−0.26
	**C2**	0.06	0.25	0.28	0.80
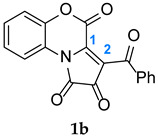	**C1**	0.25	1.16	−0.07	−0.18
	**C2**	0.09	0.42	0.25	0.64
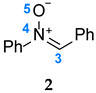	**C3**	0.21	0.35	0.16	0.58
	**O5**	0.15	0.25	0.42	1.53

**Table 3:** Energetic parameters for the 1,3-DC reactions of FPDs (**1a-b**) and diphenylnitrone (**2**) in the light of B2PLYP-D3/ma-def2-TZVPP calculations.

Reaction	Transition	ΔH [kcal/mol]	ΔG [kcal/mol]	ΔS [cal/molK]
**1a**+**2**	**1a+2**→**MC1a**	−17.5	−3.7	−55.1
	**1a+2**→**TS1a**	2.5	20.3	−59.9
	**1a+2**→**3a**	−31.0	−12.0	−60.8
	**1a+2**→**MC2a**	−18.1	−3.9	−55.2
	**1a+2**→**TS2a**	3.3	21.9	−60.5
	**1a+2**→**4a**	−30.7	−12.3	−61.2
**1b**+**2**	**1b+2**→**MC1b**	−18.9	−3.1	−54.8
	**1b+2**→**TS1b**	1.4	18.2	−59.7
	**1b+2**→**3b**	−30.1	−11.3	−59.5
	**1b+2**→**MC2b**	−19.3	−3.8	−54.1
	**1b+2**→**TS2b**	2.1	21.6	−59.8
	**1b+2**→**4b**	−36.3	−15.4	−60.2

**Table 4:** ELF valence basin populations and distances of the 1,3-DC reaction between FPDs (**1b**) and diphenylnitrone (**2**), defining the eight different phases. Distances are given in angstroms, Å, and electron populations in average number of electrons, e.

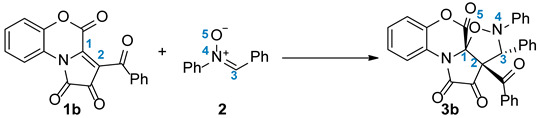																			
Points	1b	2	MC1b		P1		P2		P3		P4		P5		P6		P7		3b
Phases				*I*		*II*		*III*		*IV*		*V*		*VI*		*VII*		*VIII*	
d(C2-C3)			3.059		2.904		2.469		2.319		2.291		2.110		2.101		2.060		1.755
d(C1-O5)			2.593		2.461		2.046		1.899		1.872		1.708		1.700		1.668		1.491
V(C1,C2)	1.75		3.28		3.02		2.76		2.50		2.47		2.27		2.26		2.22		2.08
V′(C1,C2)	1.67																		
V(C3,N4)		3.75	3.65		3.64		2.87		2.45		2.38		2.06		2.05		2.00		1.79
V(N4,O5)		1.37	1.33		1.32		1.21		1.15		1.14		1.05		1.05		1.04		0.97
V(O5)		3.03	2.99		2.99		2.93		2.95		2.96		2.74		2.71		2.68		2.54
V′(O5)		2.90	2.93		2.92		2.93		2.87		2.85		2.69		2.66		2.66		2.52
V(C2)					0.27		0.60		0.75		0.78		0.93		0.94				
V(N4)							0.82		1.21		1.28		1.65		1.66		1.72		2.00
V(C1)									0.18		0.20		0.53						
V(C3)											0.24		0.40		0.41				
V″(O5)													0.17						
V(C1,O5)															0.78		0.84		1.51
V(C2,C3)																	1.40		1.73

**Table 5:** The affinities of the studied compounds.

System	Affinity (kcal/mol)
**Ligand-1**	−6.9
**Ligand-2**	−5.0
**AZT**	−3.9

**Table 6:** Predictive profile of ADMET properties for compounds **3a** and **3b** using in silico techniques.

Property	Model Name	Predicted Value of 3a	Predicted Value of 3a	Unit
Absorption	Water solubility	**−3.63**	**−3.737**	Numeric (log mol/L)
Absorption	Caco2 permeability	**1.01**	**−0.058**	Numeric (log Papp in 10^−6^ cm/s)
Absorption	Intestinal absorption (human)	**100**	**99.263**	Numeric (% Absorbed)
Absorption	Skin permeability	**−2.735**	**−2.735**	Numeric (log Kp)
Absorption	P-glycoprotein substrate	**No**	**No**	Categorical (Yes/No)
Absorption	P-glycoprotein I inhibitor	**Yes**	**Yes**	Categorical (Yes/No)
Absorption	P-glycoprotein II inhibitor	**Yes**	**Yes**	Categorical (Yes/No)
Distribution	VDss (human)	**−1.599**	**−1.715**	Numeric (log L/kg)
Distribution	Fraction unbound (human)	**0.246**	**0.16**	Numeric (Fu)
Distribution	BBB permeability	**−0.818**	**−0.462**	Numeric (log BB)
Distribution	CNS permeability	**−3.074**	**−2.056**	Numeric (log PS)
Metabolism	CYP2D6 substrate	**No**	**No**	Categorical (Yes/No)
Metabolism	CYP3A4 substrate	**Yes**	**Yes**	Categorical (Yes/No)
Metabolism	CYP1A2 inhibitor	**No**	**No**	Categorical (Yes/No)
Metabolism	CYP2C19 inhibitor	**Yes**	**Yes**	Categorical (Yes/No)
Metabolism	CYP2C9 inhibitor	**Yes**	**Yes**	Categorical (Yes/No)
Metabolism	CYP2D6 inhibitor	**No**	**No**	Categorical (Yes/No)
Metabolism	CYP3A4 inhibitor	**Yes**	**No**	Categorical (Yes/No)
Excretion	Total clearance	**−0.133**	**−0.428**	Numeric (log mL/min/kg)
Excretion	Renal OCT2 substrate	**No**	**No**	Categorical (Yes/No)
Toxicity	AMES toxicity	**No**	**No**	Categorical (Yes/No)
Toxicity	Max. tolerated dose (human)	**0.401**	**0.107**	Numeric (log mg/kg/day)
Toxicity	hERG I inhibitor	**No**	**No**	Categorical (Yes/No)
Toxicity	hERG II inhibitor	**Yes**	**Yes**	Categorical (Yes/No)
Toxicity	Oral rat acute toxicity (LD50)	**1.823**	**2.277**	Numeric (mol/kg)
Toxicity	Oral rat chronic toxicity (LOAEL)	**1.032**	**1.02**	Numeric (log mg/kg_bw/day)
Toxicity	Hepatotoxicity	**No**	**Yes**	Categorical (Yes/No)
Toxicity	Skin sensitization	**No**	**No**	Categorical (Yes/No)
Toxicity	*T. pyriformis* toxicity	**0.285**	**0.285**	Numeric (log µg/L)
Toxicity	Minnow toxicity	**−0.143**	**2.88**	Numeric (log m)

## Data Availability

The original contributions presented in this study are included in the article. Further inquiries can be directed to the corresponding author.

## Supplementary Materials

The following supporting information can be downloaded at https://www.mdpi.com/article/10.3390/molecules30183718/s1: Table S1. B3LYP(PCM)/6-311g(d,p) thermodynamic parameters for 1,3-DC reactions of FPDs (**1a-b**) and diphenylnitrone (**2**) in toluene (ΔH, ΔG in kcal/mol; ΔS in cal/mol·K); Table S2. M06-2X thermodynamic parameters for 1,3-DC reactions of FPDs (**1a-b**) and diphenylnitrone (**2**) in toluene (ΔH, ΔG in kcal/mol; ΔS in cal/mol·K); Table S3. wB97XD thermodynamic parameters for 1,3-DC reactions of FPDs (**1a-b**) and diphenylnitrone (**2**) in toluene (ΔH, ΔG in kcal/mol; ΔS in cal/mol·K); Table S4. B3LYP-D3(BJ) thermodynamic parameters for 1,3-DC reactions of FPDs (**1a-b**) and diphenylnitrone (**2**) in toluene (ΔH, ΔG in kcal/mol; ΔS in cal/mol·K); Table S5. B3LYP(PCM)/ 6-311G(d,p) optimized cartesian coordinates for the stationary points involved in the 1,3-DC reactions of FPDs (**1a-b**) and diphenylnitrone (**2**).
