# Supplementary material for: Study of 1,3-Dipolar Cycloaddition Between 4-Acyl-1H-pyrrole-2,3-diones Fused at the [e]-Side with a Heterocyclic Moiety and Diphenylnitrone: A Comprehensive MEDT, Docking Approach and MD Simulation
Source: Molecules. 2025 Sep 12;30(18):3718. doi: 10.3390/molecules30183718 (PMC12472521; doi:10.3390/molecules30183718)
Supplement: Supplementary file 1 [file molecules-30-03718-s001.zip › molecules-3855412-supplementary.pdf]

Supporting Information

Study of 1,3-Dipolar Cycloaddition Between 4-Acyl-1H-pyrrole-2,3-diones Fused at the [e]-Side with a Heterocyclic Moiety and Diphenylnitrone: A Comprehensive MEDT, Docking Approach and MD Simulation

Soukaina Ameur [1], Agnieszka Kącka-Zych [2,*], Ziad Moussa [3,*], Reem I. Alsantali [4], Abdellah Zeroual [1], Mustafa S. Alluhaibi [5], Abdulrahman A. Alsimaree [6] and Saleh A. Ahmed [5,*]

[1] Molecular Modelling and Spectroscopy Research Team, Faculty of Science, Chouaïb Doukkali University, P.O. Box 20, El Jadida 24000, Morocco; ameursoukaina8@gmail.com (S.A.); r.alsantli@tu.edu.sa (A.Z.)
[2] Cracow University of Technology, Faculty of Chemical Engineering and Technology, Department of Organic chemistry and Technology, Warszawska 24, 31-155 Cracow, Poland
[3] Department of Chemistry, College of Science, United Arab Emirates University, Al Ain P.O. Box 15551, United Arab Emirates
[4] Department of Pharmaceutical Chemistry, College of Pharmacy, Taif University, P.O. Box 11099, Taif 21944, Saudi Arabia; zeroualabdellah2@gmail.com
[5] Department of Chemistry, Faculty of Science, Umm Al-Qura University, Makkah 21955, Saudi Arabia; msluhaibi@uqu.edu.sa
[6] Department of Chemistry, College of Science and Humanities, Shaqra University, Shaqra 11911, Saudi Arabia; alsimaree@su.edu.sa
* Correspondence: agnieszka.kacka-zych@pk.edu.pl (A.K.-Z.); zmoussa@uaeu.ac.ae (Z.M.); saahmed@uqu.edu.sa (S.A.A.)

S3 **S1. Theoretical Background**

S5 **S2. Computational methodology**

S6 **Table S1.** B3LYP(PCM)/6-311g(d,p) thermodynamic parameters for 1,3-DC reactions of FPDs (**1a-b**) and diphenylnitrone (**2**) in toluene (ΔH, ΔG in kcal/mol; ΔS in cal/mol·K).

S7 **Table S2.** M06-2X thermodynamic parameters for 1,3-DC reactions of FPDs (**1a-b**) and diphenylnitrone (**2**) in toluene (ΔH, ΔG in kcal/mol; ΔS in cal/mol·K).

S7 **Table S3.** wB97XD thermodynamic parameters for 1,3-DC reactions of FPDs (**1a-b**) and diphenylnitrone (**2**) in toluene (ΔH, ΔG in kcal/mol; ΔS in cal/mol·K).

S8 **Table S4.** B3LYP-D3(BJ) thermodynamic parameters for 1,3-DC reactions of FPDs (**1a-b**) and diphenylnitrone (**2**) in toluene (ΔH, ΔG in kcal/mol; ΔS in cal/mol·K).

S9 **Table S5.** B3LYP(PCM)/ 6-311G(d,p) optimized cartesian coordinates for the stationary points involved in the 1,3-DC reactions of FPDs (**1a-b**) and diphenylnitrone (**2**).

S39 **3. References**

S1. Theoretical background

S1.1 BET studies

The so-called Bonding Evolution Theory (BET) [1] has proven to be a very useful methodological tool in order to have a better understanding of the bonding changes along a pathway, and therefore, to establish the nature of the electronic rearrangement associated with a given molecular mechanism [2-7]. Within molecular electron density theory (MEDT) scheme [8], the bonding changes are topologically and energetically analysed in order to understand the origin of the activation and the reaction energy associated to an organic reaction.

For the analyzed 1,3-dipolar cycloadditions reaction between FPDs (**1a-b**) and diphenylnitrone (**2**), the populations, among other relevant parameters, of the most significant Electron Localisation Function (ELF) [9] valence basins [10] (those associated with the bonding regions directly involved in the reaction) of the selected structures of the Intrinsic Reaction Coordinate (IRC) [11], defining the different topological phases are gathered in Table 4. A simplified representation of the molecular mechanism by ELF-based Lewis structures (Scheme 3), the basin-population changes, together with their associated energies along the reaction path and some relevant attractor positions involved in the bond formation processes are shown in Figures 5.

S1.2 Topological analysis of the electron localisation function (ELF)

In order to adjust a chemical description of matter, namely, chemical bond, with quantum chemical postulates, several mathematical models have been developed. Among them, Bader introduced through the Theory of Atoms in Molecules (AIM),[12] which has become a powerful method of analysis. The AIM theory enables a partition of the electron density within the molecular space into basins associated with atoms. Another relevant procedure that provides the connection between the electron density distribution and the chemical structure is the quantum chemical analysis of the ELF of Becke and Edgecombe.[9] ELF constitutes a useful relative measure of the electron pair localisation characterising the corresponding electron density.[13] Within the framework of Density Functional Theory (DFT),[14] ELF is a density-based property that can be interpreted in terms of the positive-definite local Pauli and Thomas Fermi kinetic energy densities in a given system. In the validity of such a framework, these quantities provide key information to evaluate the relative local excess of kinetic energy density associated to the Pauli principle. ELF presents values in the range [0,1]; the highest [13,15,16] after an analysis of the electron density, ELF provides basins of attractors, which are the domains in which the probability of finding an electron pair

is maximal. The spatial points in which the gradient of ELF has a maximum value are designated as attractors.[17-19] ELF basins are classified as core basins, C(...), and valence basins, V(...). The latter are characterised by the synaptic order, i.e. the number of atomic valence shells in which they participate. Thus, there are monosynaptic, disynaptic, trisynaptic basins and so on.[10] Monosynaptic basins, labelled V(A), correspond to the lone pairs or non-bonding regions, while disynaptic basins, labelled V(A,B), connect the core of two nuclei A and B and, thus, correspond to a bonding region between A and B. This description recovers the Lewis bonding model, providing a graphical representation of the molecular system.

S1.3 Bonding Evolution Theory (BET)

As mentioned, the so-called BET has proved to be a very useful methodological tool.[1] BET applies Thom's Catastrophe Theory (CT) concepts [20-22] to the topological analysis of the gradient field of the ELF.[9]

Within the BET methodology,[1] the structural stability of the critical points of the ELF gradient field is examined for the system of nuclei and electrons 'evolving' along the Born-Oppenheimer energy hypersurface or a given reduced reaction coordinate, e.g. the intrinsic reaction coordinate, occurring as a result of the variation in the control space parameters from reactive to product configurations. The chemical processes are rationalised in terms of successive structural stability domains (SSDs), here called phases, comprising structures along the path where the number and type, e.g. synaptic orders, of critical points of the gradient field of ELF remain without changes.[1]

Within the BET context, the turning points between these phases are located and the discontinuities or bifurcation catastrophes can be identified. Hence BET allows to characterize unequivocally the behavior of the dynamical system upon bifurcations associated with the changes of the ELF gradient field along the reaction coordinate. The different catastrophes, in this case, correspond to the reduction or the increase of the critical points associated with attractors of electron pairs defining bonding and non-bonding, e.g. lone pairs, domains for electron (de)localisation.

Many theoretical studies have shown that the topological analysis of the ELF offers a suitable framework for the study of the changes of electron density.[23-35] This methodological approach is used as a valuable tool to understand the bonding changes along the reaction path and, consequently, to establish the nature of the electronic rearrangement associated with a given molecular mechanism within a BET perspective.

S2. Computational methodology

The computational study was performed using the B3LYP(PCM)/6-311G(d,p) level of theory with the Gaussian 16 package [36]. The PLGrid infrastructure ("Ares" supercomputer) at the national computing centre "Cyfronet" was utilized. All optimised critical points were verified on the basis of the full vibrational analysis. Next, intrinsic reaction coordinate (IRC) calculations were performed for all optimized transition states. The obtained IRC trajectories confirmed, without doubt, the postulated nature of the TSs and their role within the energy profile. The presence of solvent (toluene) in the reaction environment was included using the IEFPCM (Integral Equation Formalism Polarizable Continuum Model) algorithm [37]. Calculations of all critical structures were performed at temperature T=298K and pressure p=1atm.

The global electron density transfer (GEDT) [38,39] within critical structures was estimated using the formula:

$$GEDT= -\Sigma qA$$

where qA is the net charge, and the sum is taken over all the atoms of diphenylonitrone.

Global and local electronic properties of the reactants were estimated using equations recommended by Parr and Domingo [40,41]. In particular, the electronic chemical potentials (μ) and chemical hardness (η) were evaluated in terms of one-electron energies of the frontier molecular orbitals (HOMO and LUMO) using the following equations:

$$\mu \approx (EHOMO + ELUMO)/2 \qquad \eta \approx ELUMO – EHOMO$$

The values of μ and η were then used to calculate the global electrophilicity index (ω) using the formula:

$$\omega=\mu^2/2\eta$$

Global nucleophilicity (N) [42] was expressed using the equation:

$$N=E_{HOMO} – E_{HOMO(tetracyanoethene)}$$

The local electrophilicity (ω_k) at atom k was calculated by projecting the index ω into any reaction centre k in the molecule using Parr functions P_k^+ [43]:

$$\omega_k = P^+_k \cdot \omega$$

The local nucleophilicity (Nk) condensed to atom k was calculated using global nucleophilicity N and Parr functions P_k- [43] according to the formula:

$$N_k = P^{-}_{k} \cdot N$$

The ELF [9] studies were performed with the TopMod package [44] considering the standard cubical grid of step size of 0.1 Bohr. The bonding changes along corresponding reactions were analyzed, according to the BET [1], by performing the topological analysis of the ELF for 200 nuclear configurations for reaction leading to **MC1b** and 400 nuclear configurations leading to product **3b**. The ELF molecular geometries and basin attractor positions were visualized using the GaussView program [45]. ELF localization domains were represented by using the Paraview software at an isovalue of 0.80 a.u [46,47].

Table S1. B3LYP(PCM)/6-311G(d,p) thermodynamic parameters for 1,3-DC reactions of FPDs (**1a-b**) and diphenylnitrone (**2**) in toluene (ΔH, ΔG in kcal/mol; ΔS in cal/mol·K).

**Reaction**	**Transition**	**ΔH [kcal/mol]**	**ΔG [kcal/mol]**	**ΔS [cal/molK]**
**1a+2**	1a+2→MC1a	-3.7	5.6	-31.1
	1a+2→TS1a	26.2	41.6	-51.5
	1a+2→3a	1.3	17.8	-55.4
	1a+2→MC2a	-4.2	5.9	-33.9
	1a+2→TS2a	27.7	43.1	-51.9
	1a+2→4a	2.7	17.9	-51.1
	1a+2→MC3a	-1.2	6.7	-28.4
	1a+2→TS3a	33.8	48.7	-50.2
	1a+2→5a	3.1	21.4	-54.3
	1a+2→MC4a	-1.9	7.1	-26.9
	1a+2→TS4a	32.5	52.2	-49.7
	1a+2→6a	3.6	21.5	-55.1
**1b+2**	1b+2→MC1b	-5.1	5.2	-34.5
	1b+2→TS1b	24.7	40.1	-51.7
	1b+2→3b	0.3	17.1	-56.2
	1b+2→MC2b	-5.3	5.8	-35.8
	1b+2→TS2b	24.9	40.9	-53.5
	1b+2→4b	0.6	16.7	-53.8
	1b+2→MC3b	-4.3	7.1	-29.4
	1b+2→TS3b	26.5	46.2	-56.2
	1b+2→5b	0.9	19.2	-58.1
	1b+2→MC4b	-4.1	6.8	-30.5
	1b+2→TS4b	26.9	45.9	-55.8
	1b+2→6b	1.2	17.8	-56.9

Table S2. M06-2X thermodynamic parameters for 1,3-DC reactions of FPDs (**1a-b**) and diphenylnitrone (**2**) in toluene (ΔH, ΔG in kcal/mol; ΔS in cal/mol·K).

Basic set	Reaction	Transition	ΔH	ΔG	ΔS
6-311+G(d,p)	1a+2	**1a+2→MC1a**	-17.2	-3.4	-46.4
		**1a+2→TS1a**	2.4	20.5	-60.6
		**1a+2→3a**	-29.4	-11.4	-60.4
		**1a+2→MC2a**	-16.8	-2.1	-45.8
		**1a+2→TS2a**	2.9	21.2	-60.9
		**1a+2→4a**	-30.4	-12.3	-59.9
	1b+2	**1b+2→MC1b**	-19.1	-2.9	-54.3
		**1b+2→TS1b**	0.7	18.5	-59.8
		**1b+2→3b**	-30.3	-11.8	-61.8
		**1b+2→MC2b**	-18.9	-2.6	-55.2
		**1b+2→TS2b**	1.1	18.8	-60.3
		**1b+2→4b**	-29.8	-11.1	-60.1
6-311++G(d,p)	1a+2	**1a+2→MC1a**	-17.5	-3.6	-54.2
		**1a+2→TS1a**	2.2	20.2	-60.6
		**1a+2→3a**	-30.5	-12.0	-61.7
		**1a+2→MC2a**	-18.1	-3.9	-53.2
		**1a+2→TS2a**	3.4	22.1	-61.5
		**1a+2→4a**	-31.2	-12.4	-60.8
	1b+2	**1b+2→MC1b**	-19.3	-3.1	-54.2
		**1b+2→TS1b**	0.9	18.3	-60.1
		**1b+2→3b**	-30.5	-12.0	-61.7
		**1b+2→MC2b**	-19.5	-3.4	-55.2
		**1b+2→TS2b**	2.4	21.7	-61.4
		**1b+2→4b**	-37.3	-15.6	-63.0

Table S3. wB97XD thermodynamic parameters for 1,3-DC reactions of FPDs (**1a-b**) and diphenylnitrone (**2**) in toluene (ΔH, ΔG in kcal/mol; ΔS in cal/mol·K).

Basic set	Reaction	Transition	ΔH	ΔG	ΔS
6-311+G(d,p)	1a+2	**1a+2→MC1a**	-17.3	-4.4	-43.2
		**1a+2→TS1a**	5.3	23.5	-60.9
		**1a+2→3a**	-27.1	-9.8	--59.3
		**1a+2→MC2a**	-18.2	-3.5	-43.7
		**1a+2→TS2a**	6.0	24.2	-61.4
		**1a+2→4a**	-28.9	-10.5	-58.5
	1b+2	**1b+2→MC1b**	-18.9	-2.7	-53.8
		**1b+2→TS1b**	3.7	21.4	-59.3
		**1b+2→3b**	-28.0	-10.2	-59.6
		**1b+2→MC2b**	-18.1	-2.4	-54.7
		**1b+2→TS2b**	3.9	21.6	-59.9
		**1b+2→4b**	-27.6	-10.0	-58.9
6-311++G(d,p)	1a+2	**1a+2→MC1a**	-19.8	-4.6	-54.9

		**1a+2→TS1a**	5.1	23.2	-60.8
		**1a+2→3a**	-27.5	-9.5	-60.3
		**1a+2→MC2a**	-19.0	-3.8	-48.7
		**1a+2→TS2a**	5.8	24.0	-60.9
		**1a+2→4a**	-32.4	-11.2	-59.3
	**1b+2**	**1b+2→MC1b**	-19.0	-4.2	-49.6
		**1b+2→TS1b**	3.9	20.9	-60.1
		**1b+2→3b**	-28.2	-10.5	-59.7
		**1b+2→MC2b**	-18.7	-4.3	-49.3
		**1b+2→TS2b**	5.0	23.3	-60.3
		**1b+2→4b**	-33.1	-13.2	-59.7

Table S4. B3LYP-D3(BJ) thermodynamic parameters for 1,3-DC reactions of FPDs (**1a-b**) and diphenylnitrone (**2**) in toluene (ΔH, ΔG in kcal/mol; ΔS in cal/mol·K).

Basic set	Reaction	Transition	ΔH	ΔG	ΔS
6-311+G(d,p)	**1a+2**	**1a+2→MC1a**	-16.9	-3.2	-48.3
		**1a+2→TS1a**	2.6	20.8	-59.8
		**1a+2→3a**	-27.9	-10.7	-59.7
		**1a+2→MC2a**	-17.1	-2.3	-46.3
		**1a+2→TS2a**	3.1	21.5	-60.4
		**1a+2→4a**	-30.9	-13.0	-60.1
	**1b+2**	**1b+2→MC1b**	-19.4	-3.0	-55.1
		**1b+2→TS1b**	0.9	18.9	-60.1
		**1b+2→3b**	-31.0	-12.7	-60.3
		**1b+2→MC2b**	-19.1	-2.9	-54.8
		**1b+2→TS2b**	1.5	19.4	-59.7
		**1b+2→4b**	-30.6	-11.3	-59.9
6-311++G(d,p)	**1a+2**	**1a+2→MC1a**	-17.7	-3.8	-54.8
		**1a+2→TS1a**	2.4	20.5	-60.2
		**1a+2→3a**	-30.9	-12.2	-61.1
		**1a+2→MC2a**	-18.6	-4.0	-54.7
		**1a+2→TS2a**	3.7	22.3	-60.9
		**1a+2→4a**	-30.9	-12.7	-60.3
	**1b+2**	**1b+2→MC1b**	-19.9	-3.6	-55.3
		**1b+2→TS1b**	1.5	18.5	-59.9
		**1b+2→3b**	-30.8	-12.2	-60.4
		**1b+2→MC2b**	-19.7	-4.0	-53.9
		**1b+2→TS2b**	2.7	22.1	-60.4
		**1b+2→4b**	-37.9	-16.1	-61.3

Table S5. B3LYP(PCM)/ 6-311G(d,p) optimized cartesian coordinates for the stationary points involved in the 1,3-DC reactions of FPDs (**1a-b**) and diphenylnitrone (**2**).

**1a**	C	-5.15782700	-1.46842500	0.33738300
	C	-3.98795700	-2.08619000	-0.08785600
	C	-2.80538600	-1.35350200	-0.18643500
	C	-2.79767000	0.01223500	0.14569100
	C	-3.97622700	0.62834100	0.57276200
	C	-5.14910800	-0.11461000	0.66613100
	H	-1.65104200	-2.95322400	-0.86002600
	H	-6.07169100	-2.04491700	0.41016400
	H	-3.98232900	-3.13890000	-0.34765100
	H	-3.96382100	1.67735300	0.82489500
	H	-6.05772100	0.37246900	0.99763900
	C	-0.38037200	0.06889600	-0.39559100
	C	-0.39960900	-1.37441000	-0.75138600
	C	-1.29829200	2.05478500	0.26331100
	C	0.22592700	2.22602500	-0.07005700
	C	0.69578200	0.89251600	-0.45820000
	N	-1.56935200	0.69978700	0.02581900
	N	-1.62116500	-1.97094800	-0.61631800
	O	0.60022500	-1.95592000	-1.13054300
	O	0.80441400	3.27885700	0.01308800
	O	-2.05577400	2.90572900	0.64184800
	C	2.07888800	0.61848900	-0.98973500
	C	3.07905400	-0.04577100	-0.11240200
	C	4.34979400	-0.31742600	-0.63880500
	C	2.80015200	-0.38054000	1.21756600
	C	5.31969200	-0.91953800	0.15056800
	H	4.55234100	-0.04880500	-1.66795000
	C	3.77551800	-0.97871100	2.00986600
	H	1.82421800	-0.16761200	1.63641000
	C	5.03409600	-1.25057500	1.47706500
	H	6.29913300	-1.13119200	-0.26247400
	H	3.55517400	-1.23152000	3.04044200
	H	5.79311700	-1.71851300	2.09379800
	O	2.35082400	1.02301600	-2.10327200
**1b**	C	-5.12438200	-1.50093400	0.32658500
	C	-3.94032400	-2.09820600	-0.09280300
	C	-2.78692000	-1.33367700	-0.17894700
	C	-2.79123100	0.02560100	0.14820500
	C	-3.98381400	0.62137500	0.56908100
	C	-5.14084700	-0.14627400	0.65479500
	H	-6.02836300	-2.09297400	0.39491200
	H	-3.88839200	-3.14658700	-0.35741900
	H	-3.99094700	1.67089900	0.82107500
	H	-6.06068300	0.32309400	0.98101000
	C	-0.39354600	0.07203900	-0.38944000
	C	-0.43687900	-1.37125600	-0.73327800
	C	-1.29298300	2.06985200	0.26760000

	C	0.23284600	2.22379000	-0.06503700
	C	0.69203500	0.87887200	-0.45617800
	N	-1.57151000	0.72049200	0.03118800
	O	0.52617300	-1.98007000	-1.10571300
	O	0.82929800	3.26456600	0.01492200
	O	-2.04510800	2.92655100	0.64423300
	C	2.07126400	0.59503900	-0.99728500
	C	3.07841300	-0.05385200	-0.12145300
	C	4.34682500	-0.32656500	-0.65410600
	C	2.80708800	-0.37841900	1.21306600
	C	5.32235900	-0.91905100	0.13499700
	H	4.54278600	-0.06673200	-1.68675700
	C	3.78837000	-0.96780700	2.00419300
	H	1.83316200	-0.16528500	1.63675900
	C	5.04448000	-1.24006300	1.46576300
	H	6.30002300	-1.13166400	-0.28141800
	H	3.57456500	-1.21394500	3.03759800
	H	5.80806600	-1.70136800	2.08171300
	O	2.31998900	0.97658500	-2.12381600
	O	-1.63612400	-1.99034200	-0.60716600
**2**	C	0.44874900	-0.40802700	0.12406900
	O	-0.24551200	1.74107500	-0.34910200
	H	0.07086600	-1.38532500	0.38462300
	N	-0.47566000	0.50648000	-0.10352400
	C	1.88207200	-0.22332900	0.07447900
	C	2.67827200	-1.35534100	0.35416000
	C	2.52922100	0.99210000	-0.23131400
	C	4.06276500	-1.28047600	0.32763600
	H	2.19627700	-2.29787900	0.59251700
	C	3.91882200	1.05425900	-0.25728700
	H	1.92685800	1.86187200	-0.44075500
	C	4.69118900	-0.07214700	0.02032000
	H	4.65442900	-2.16227400	0.54581000
	H	4.40244500	1.99512300	-0.49529600
	H	5.77334600	-0.01132700	-0.00089500
	C	-1.88575600	0.12701500	-0.04569800
	C	-2.33198200	-1.10503600	-0.51912500
	C	-2.77826800	1.06661100	0.46416200
	C	-3.69032000	-1.40752100	-0.44962400
	H	-1.64076500	-1.80794300	-0.96635600
	C	-4.13062500	0.75098500	0.53431700
	H	-2.39744500	2.02574100	0.78649600
	C	-4.58995800	-0.48602700	0.08165800
	H	-4.04391300	-2.35997100	-0.82660000
	H	-4.82764000	1.47468800	0.94030500
	H	-5.64581200	-0.72506000	0.13111900
**MC1a**	C	-3.93304800	-0.39493200	2.96738000
	C	-2.64281400	0.07592200	3.18125100
	C	-1.56664500	-0.49484000	2.50247500
	C	-1.78725100	-1.54736200	1.59743700

C	-3.08563600	-2.01629400	1.38583400
C	-4.15069600	-1.43911500	2.07125400
H	-0.12660800	0.73115800	3.37390400
H	-4.76225700	0.05281000	3.50137600
H	-2.45883700	0.88835900	3.87549100
H	-3.24794900	-2.82394000	0.68926900
H	-5.15304900	-1.81189400	1.89989500
C	0.64152500	-1.57549000	1.12824900
C	0.86339000	-0.45918200	2.08180800
C	-0.60439600	-3.13160000	0.01923500
C	0.91312200	-3.25993500	-0.35595900
C	1.59550500	-2.21042200	0.40280200
N	-0.65866900	-2.08851000	0.94843100
N	-0.26322100	-0.03254800	2.72393400
O	1.96997700	0.01269500	2.27553900
O	1.33214700	-4.08409700	-1.12973300
O	-1.51149600	-3.79400000	-0.40856000
C	-1.36699700	2.64843700	-1.04025200
O	-0.27979700	0.65277500	-0.62767900
H	-2.19783000	3.06570000	-1.59051500
N	-1.23824400	1.34199700	-1.14366500
C	-0.52602100	3.55734400	-0.29240200
C	-0.90006000	4.91877200	-0.30828600
C	0.62303900	3.18556000	0.43591600
C	-0.16247100	5.87036400	0.37994400
H	-1.77882600	5.22220000	-0.86764700
C	1.35530700	4.14876300	1.12277900
H	0.92849300	2.15203100	0.44653400
C	0.97040600	5.48816200	1.10117800
H	-0.46740000	6.91022300	0.35487100
H	2.23679800	3.84496300	1.67570800
H	1.54928800	6.23089300	1.63816200
C	-2.21585800	0.58500900	-1.91880200
C	-3.57596600	0.87665400	-1.84559300
C	-1.74625700	-0.46658400	-2.70190800
C	-4.47358100	0.11660700	-2.59244400
H	-3.93654800	1.66075900	-1.19203000
C	-2.65132300	-1.21606400	-3.44528400
H	-0.68691100	-0.68232700	-2.71800300
C	-4.01470600	-0.92541300	-3.39503600
H	-5.53383900	0.33165900	-2.53118600
H	-2.29061400	-2.03203500	-4.05982300
H	-4.71703800	-1.51747000	-3.96975500
C	3.09386200	-2.06655000	0.43505300
C	3.74629100	-0.97473500	-0.33635000
C	5.14788200	-0.92815000	-0.35920900
C	3.01197200	-0.01649100	-1.04534300
C	5.80417400	0.06016500	-1.07951500
H	5.69965900	-1.67779700	0.19401900
C	3.67448100	0.97408900	-1.76622200

	H	1.92869900	-0.02876700	-1.03002300
	C	5.06717200	1.01380200	-1.78564900
	H	6.88765800	0.09096700	-1.09415900
	H	3.10113800	1.71677600	-2.30884500
	H	5.57959300	1.78614600	-2.34856500
	O	3.73178200	-2.91686200	1.02723100
TS1a	C	-5.57133900	-2.34679700	0.43493000
	C	-4.37158200	-2.86159500	0.91252800
	C	-3.15969700	-2.30520200	0.50397300
	C	-3.15417800	-1.21885800	-0.38601300
	C	-4.36308200	-0.70075200	-0.85792600
	C	-5.56552400	-1.26952600	-0.44783400
	H	-1.96184400	-3.57743400	1.62831400
	H	-6.50759300	-2.78938700	0.75324000
	H	-4.36514900	-3.69959500	1.60113700
	H	-4.34670300	0.12366700	-1.55337400
	H	-6.49793600	-0.86665200	-0.82394500
	C	-0.70319400	-1.00788900	-0.05799600
	C	-0.71365100	-2.20297000	0.85973900
	C	-1.63361400	0.06818800	-1.89725600
	C	-0.09502200	0.21505000	-1.94827800
	C	0.45419300	-0.49765100	-0.77134600
	N	-1.89225800	-0.72967300	-0.78478900
	N	-1.94299000	-2.81310700	0.96590100
	O	0.23791700	-2.56597700	1.51268000
	O	0.45001400	0.80308000	-2.85275700
	O	-2.42573900	0.53283400	-2.67363700
	C	0.91495200	1.24472000	0.54574100
	O	-0.84591000	0.18378400	1.45975700
	H	1.17939200	2.02494800	-0.15774800
	N	-0.36872000	1.32332000	0.99027000
	C	2.02782300	0.82364600	1.41468100
	C	3.26152600	1.46345500	1.20128500
	C	1.91354600	-0.08451300	2.47972000
	C	4.34849400	1.21315500	2.02868100
	H	3.36562100	2.16696500	0.38371000
	C	3.00515200	-0.32785100	3.30474000
	H	0.98485500	-0.60521100	2.64368300
	C	4.22298200	0.31399100	3.08503500
	H	5.29017800	1.71756700	1.84670000
	H	2.90381700	-1.03413700	4.12060500
	H	5.07016700	0.11092000	3.73044300
	C	-1.29140300	2.35612900	0.63008200
	C	-0.84390000	3.59140600	0.14551000
	C	-2.65543700	2.15554300	0.86605400
	C	-1.76539800	4.58965800	-0.15290200
	H	0.21083000	3.79429200	0.01787500
	C	-3.56155000	3.16796300	0.57485700
	H	-2.98569000	1.21311700	1.27677300
	C	-3.12690500	4.38578800	0.05565200

	H	-1.40911300	5.53769000	-0.53863400
	H	-4.61656700	2.99911100	0.75700900
	H	-3.83829400	5.17034200	-0.17232900
	C	1.63842300	-1.43296200	-1.00206000
	C	3.02438000	-0.93155500	-1.28055000
	C	4.07636400	-1.78776800	-0.92240500
	C	3.31758100	0.27437000	-1.92612200
	C	5.39383700	-1.43288200	-1.17982700
	H	3.83695500	-2.72774100	-0.44200700
	C	4.63902700	0.61053000	-2.21315100
	H	2.51513400	0.91879600	-2.25752600
	C	5.67920800	-0.23290900	-1.83187000
	H	6.19867800	-2.09540100	-0.88200800
	H	4.85339900	1.53298000	-2.74152500
	H	6.70684100	0.03766800	-2.04851400
	O	1.43231100	-2.62969200	-0.98949000
**3a**	C	-5.53803100	-2.38295600	0.44609400
	C	-4.33418100	-2.87221400	0.94121800
	C	-3.12986300	-2.29167300	0.54294300
	C	-3.13240100	-1.21115100	-0.35176900
	C	-4.34625300	-0.71830300	-0.83993100
	C	-5.54284900	-1.30853500	-0.44122600
	H	-1.91992500	-3.56620900	1.65219100
	H	-6.46922500	-2.84174200	0.75580900
	H	-4.31929600	-3.70640000	1.63392000
	H	-4.34003100	0.10614500	-1.53546400
	H	-6.47843900	-0.92351900	-0.82763700
	C	-0.72187700	-0.80100600	0.17512400
	C	-0.70217000	-2.12263800	0.97631100
	C	-1.60579800	0.02628000	-1.87321300
	C	-0.08178700	0.21690600	-1.91575000
	C	0.53022100	-0.30181600	-0.60207800
	N	-1.86540100	-0.70180400	-0.73282700
	N	-1.90574300	-2.77346600	1.02253900
	O	0.25386900	-2.47384500	1.62785300
	O	0.46348500	0.71454900	-2.86488200
	O	-2.38588800	0.43677700	-2.69836200
	C	0.93857400	1.11383200	0.41571400
	O	-0.88989800	0.13968600	1.31930200
	H	1.22752000	1.91980200	-0.25597900
	N	-0.34220600	1.39154800	1.00297800
	C	2.04242100	0.85986000	1.43555200
	C	3.29862100	1.43910500	1.21587500
	C	1.87412800	0.04450600	2.56571900
	C	4.37371600	1.17151500	2.06100500
	H	3.44722400	2.09015400	0.36229000
	C	2.94930900	-0.22131100	3.41009300
	H	0.91729400	-0.40982100	2.76747100
	C	4.20485000	0.32821200	3.15570000
	H	5.34005200	1.61869200	1.85813100

	H	2.80406800	-0.87115400	4.26559400
	H	5.04164200	0.10532700	3.80807000
	C	-1.25796400	2.40351100	0.60168400
	C	-0.79116200	3.66214100	0.18771400
	C	-2.63902700	2.20614200	0.74438200
	C	-1.69750700	4.67134000	-0.13171800
	H	0.27128300	3.86431200	0.13071600
	C	-3.52914000	3.22694200	0.42683800
	H	-3.00744600	1.25848000	1.10762400
	C	-3.07068600	4.46301800	-0.02289400
	H	-1.31761800	5.63214700	-0.46027400
	H	-4.59273000	3.04685600	0.53589000
	H	-3.76866200	5.25312800	-0.27180500
	C	1.64223600	-1.38722800	-0.91074600
	C	3.02061300	-0.93794000	-1.27607600
	C	4.07183500	-1.82621900	-0.99802600
	C	3.30658600	0.28150300	-1.89936800
	C	5.38159300	-1.48503000	-1.30955000
	H	3.84387500	-2.77248900	-0.52453600
	C	4.61663100	0.60536200	-2.23947800
	H	2.50843700	0.95815900	-2.16515300
	C	5.65734800	-0.26807700	-1.93480300
	H	6.18784200	-2.16847400	-1.06976700
	H	4.82248200	1.54340700	-2.74209900
	H	6.67868200	-0.00592700	-2.18704400
	O	1.38386500	-2.57089900	-0.86359000
	C	-5.04109700	-2.18404100	-1.06050500
	C	-3.79159000	-2.77687600	-1.21087700
	C	-2.64284800	-1.98711900	-1.24986400
	C	-2.75197900	-0.59076200	-1.13641300
	C	-4.00622200	-0.00217900	-0.96149000
	C	-5.14675500	-0.80106200	-0.93126200
	H	-1.31472500	-3.56830400	-1.53022900
	H	-5.92835100	-2.80526600	-1.04294300
	H	-3.69973600	-3.85303400	-1.30780000
	H	-4.07971500	1.07188300	-0.88287900
	H	-6.11738600	-0.33634800	-0.80936300
**MC2a**	C	-0.26698900	-0.45721200	-1.12931100
	C	-0.16563300	-1.93299400	-1.24085800
	C	-1.42018500	1.47537300	-1.62938100
	C	0.12020400	1.72315700	-1.65556500
	C	0.74740100	0.44904300	-1.27931900
	N	-1.55880800	0.15380600	-1.23273700
	N	-1.37714000	-2.56617300	-1.40653800
	O	0.88598800	-2.53913300	-1.22516100
	O	0.62317200	2.74747000	-2.04781500
	O	-2.28690500	2.25607900	-1.93045400
	N	-0.19553500	0.70622400	1.41404100
	C	-1.28445400	1.59699400	1.75455400
	C	-1.19703500	2.93614400	1.37312600

	C	-2.35361500	1.14291500	2.52943000
	C	-2.21468100	3.81133100	1.73667500
	H	-0.34207000	3.27639800	0.80795100
	C	-3.36653200	2.02632300	2.87777400
	H	-2.38859700	0.12804300	2.89877000
	C	-3.30337300	3.36030300	2.48064200
	H	-2.15437000	4.84967600	1.43432200
	H	-4.19526400	1.67326300	3.47955600
	H	-4.09164800	4.04808800	2.76249500
	C	2.15426800	0.13465600	-1.71822600
	C	3.38010400	0.62922800	-1.01889700
	C	4.54914100	-0.12539100	-1.20481100
	C	3.44818000	1.82931600	-0.30054100
	C	5.75694100	0.29767700	-0.66633100
	H	4.48709700	-1.04213000	-1.77718700
	C	4.66879500	2.26517300	0.21360800
	H	2.56090200	2.42992000	-0.16402700
	C	5.82049900	1.50004900	0.04018100
	H	6.64983400	-0.30132300	-0.80470100
	H	4.71802400	3.20536400	0.75093500
	H	6.76504700	1.83999100	0.45029700
	O	2.25197000	-0.50974500	-2.74286000
	O	0.89326300	1.24550600	1.01297900
	H	-1.37707100	-0.93783300	1.57794000
	C	-0.35474500	-0.61509700	1.45016100
	C	0.66627900	-1.60162100	1.71908000
	C	0.19793000	-2.89616100	2.02039100
	C	2.03729000	-1.32314900	1.85601100
	C	1.06125600	-3.86811100	2.49962300
	H	-0.85797100	-3.11996800	1.91056900
	C	2.89116900	-2.30045000	2.35082300
	H	2.40978100	-0.34341600	1.59947200
	C	2.41116900	-3.56752800	2.68111300
	H	0.68327900	-4.85395700	2.74423700
	H	3.94306700	-2.07157100	2.47596100
	H	3.08745400	-4.32056700	3.06886700
**TS2a**	C	-5.55523800	-2.40280400	0.43445200
	C	-4.35139100	-2.89160300	0.92990800
	C	-3.14693200	-2.31099100	0.53398800
	C	-3.15098500	-1.23007300	-0.36101000
	C	-4.36373200	-0.73750700	-0.84980000
	C	-5.56013000	-1.32830500	-0.45227300
	H	-1.94368700	-3.55444600	1.68268000
	H	-6.48625200	-2.86280600	0.74287100
	H	-4.33651200	-3.72618900	1.62210900
	H	-4.35677900	0.08388500	-1.54890400
	H	-6.49564800	-0.94487600	-0.84062500
	C	-0.70511000	-0.94996500	0.01040200
	C	-0.71397900	-2.15190300	0.93889500
	C	-1.62617700	0.01772000	-1.89568800

	C	-0.08847100	0.16431100	-1.95015100
	C	0.45801400	-0.50162100	-0.75689200
	N	-1.89262300	-0.72310800	-0.75215000
	N	-1.92532600	-2.78928400	1.02017000
	O	0.23395300	-2.48076400	1.61716500
	O	0.44924300	0.70617600	-2.89208500
	O	-2.41221300	0.43458600	-2.70802500
	N	-0.36244600	1.37541100	0.96943400
	C	-1.27735400	2.40708600	0.58963900
	C	-0.80841300	3.66125500	0.17865800
	C	-2.65137900	2.18975500	0.73069900
	C	-1.71699600	4.66267900	-0.14805100
	H	0.25068000	3.87596500	0.13238600
	C	-3.54549500	3.20606100	0.41432000
	H	-3.00452200	1.23452700	1.08860600
	C	-3.08783700	4.44260900	-0.03665400
	H	-1.34405000	5.62572500	-0.47615700
	H	-4.60845200	3.02479100	0.52217600
	H	-3.78963500	5.22949300	-0.28503300
	C	1.62843900	-1.44829600	-0.95222000
	C	3.00672000	-0.96525700	-1.29427000
	C	4.06345500	-1.84487700	-1.01181600
	C	3.28974200	0.25787800	-1.91228100
	C	5.37262200	-1.50112800	-1.32168000
	H	3.83478000	-2.79446700	-0.54562300
	C	4.60100600	0.58671100	-2.24994800
	H	2.48420200	0.92587100	-2.18142600
	C	5.64518100	-0.28393300	-1.94781100
	H	6.18039400	-2.18372300	-1.08346000
	H	4.80473600	1.52531000	-2.75314800
	H	6.66539400	-0.02011100	-2.20364900
	O	1.42553500	-2.64576600	-0.86277100
	O	0.93430500	1.31713300	0.58369400
	H	-1.77036309	0.00147795	0.89174884
	C	-0.85562000	0.21472500	1.40426300
	C	-1.12448673	0.30603296	2.91785923
	C	-2.34789058	-0.11875651	3.43591818
	C	-0.14400835	0.81321156	3.77099573
	C	-2.59117329	-0.03615675	4.80721765
	H	-3.12060310	-0.51882123	2.76360397
	C	-0.38733949	0.89626252	5.14191859
	H	0.82065133	1.14768146	3.36229556
	C	-1.61146889	0.47175910	5.66010833
	H	-3.55607196	-0.37041944	5.21523825
	H	0.38499220	1.29664601	5.81459471
	H	-1.80338953	0.53747958	6.74091534
**4a**	C	-5.03458900	-2.18474000	-1.06690900
	C	-3.78389200	-2.77544400	-1.21934900
	C	-2.63895400	-1.97903600	-1.25121700
	C	-2.75341000	-0.58767400	-1.10128500

C	-4.01032600	0.00050000	-0.94796500
C	-5.14791100	-0.80150000	-0.93745700
H	-1.30202600	-3.52283100	-1.67221000
H	-5.92008900	-2.80868700	-1.05140700
H	-3.69012300	-3.85038200	-1.32654700
H	-4.08347600	1.07302500	-0.85132100
H	-6.12170500	-0.34218600	-0.82126500
C	-0.29699400	-0.50545400	-0.64997300
C	-0.15804400	-1.93232000	-1.19493900
C	-1.40568500	1.43324100	-1.60478500
C	0.09817200	1.70855700	-1.61370100
C	0.77401000	0.54702200	-0.91488500
N	-1.54653500	0.14316800	-1.13095400
N	-1.36700000	-2.53874600	-1.44473500
O	0.89283500	-2.52669100	-1.26333200
O	0.62136300	2.69037400	-2.07029500
O	-2.26853800	2.19900600	-1.96417600
N	-0.15400400	0.71904700	1.40366400
C	-1.23257800	1.60059400	1.71664400
C	-1.17915000	2.95524200	1.37127400
C	-2.31021000	1.13212900	2.49089800
C	-2.21297100	3.81071500	1.74448300
H	-0.33458000	3.33332700	0.81278000
C	-3.33237200	2.00206000	2.86014400
H	-2.34078400	0.10262700	2.82523200
C	-3.29695400	3.34436000	2.48366500
H	-2.16232500	4.85283700	1.45096800
H	-4.15704400	1.62425300	3.45388400
H	-4.09496700	4.01736200	2.77320800
C	2.13173800	0.17005500	-1.59922800
C	3.39512400	0.64117000	-0.99454700
C	4.55149600	-0.12459300	-1.20091000
C	3.47202400	1.84750300	-0.29028800
C	5.76324700	0.29634400	-0.67011800
H	4.47718600	-1.04701200	-1.76319800
C	4.69336200	2.27578000	0.21642800
H	2.59394700	2.46117200	-0.15851900
C	5.83470500	1.49720900	0.03922400
H	6.65376100	-0.30491300	-0.81155300
H	4.75324700	3.21709900	0.74985500
H	6.78287400	1.82750400	0.44823100
O	2.10266800	-0.44298600	-2.64444200
O	0.89078800	1.19795400	0.64459700
H	-1.35824400	-0.96766600	1.24731300
C	-0.34875900	-0.64593200	0.99453700
C	0.64784500	-1.62176300	1.61395000
C	0.18021600	-2.88597600	1.98764500
C	2.00123900	-1.32258200	1.82785400
C	1.04165000	-3.84637600	2.51432600
H	-0.86947100	-3.13057800	1.85833500

	C	2.86062200	-2.28111500	2.36032700
	H	2.38591400	-0.34512400	1.57534800
	C	2.38988700	-3.55029800	2.69248800
	H	0.65603200	-4.82249400	2.78583500
	H	3.90572600	-2.03380500	2.50786700
	H	3.06530500	-4.29694200	3.09433200
**MC3a**	C	-3.93304800	-0.39493200	2.96738000
	C	-2.64281400	0.07592200	3.18125100
	C	-1.56664500	-0.49484000	2.50247500
	C	-1.78725100	-1.54736200	1.59743700
	C	-3.08563600	-2.01629400	1.38583400
	C	-4.15069600	-1.43911500	2.07125400
	H	-0.12660800	0.73115800	3.37390400
	H	-4.76225700	0.05281000	3.50137600
	H	-2.45883700	0.88835900	3.87549100
	H	-3.24794900	-2.82394000	0.68926900
	H	-5.15304900	-1.81189400	1.89989500
	C	0.64152500	-1.57549000	1.12824900
	C	0.86339000	-0.45918200	2.08180800
	C	-0.60439600	-3.13160000	0.01923500
	C	0.91312200	-3.25993500	-0.35595900
	C	1.59550500	-2.21042200	0.40280200
	N	-0.65866900	-2.08851000	0.94843100
	N	-0.26322100	-0.03254800	2.72393400
	O	1.96997700	0.01269500	2.27553900
	O	1.33214700	-4.08409700	-1.12973300
	O	-1.51149600	-3.79400000	-0.40856000
	C	-1.36699700	2.64843700	-1.04025200
	O	-0.27979700	0.65277500	-0.62767900
	N	-1.23824400	1.34199700	-1.14366500
	C	-2.21585800	0.58500900	-1.91880200
	C	-3.57596600	0.87665400	-1.84559300
	C	-1.74625700	-0.46658400	-2.70190800
	C	-4.47358100	0.11660700	-2.59244400
	H	-3.93654800	1.66075900	-1.19203000
	C	-2.65132300	-1.21606400	-3.44528400
	H	-0.68691100	-0.68232700	-2.71800300
	C	-4.01470600	-0.92541300	-3.39503600
	H	-5.53383900	0.33165900	-2.53118600
	H	-2.29061400	-2.03203500	-4.05982300
	H	-4.71703800	-1.51747000	-3.96975500
	C	3.09386200	-2.06655000	0.43505300
	C	3.74629100	-0.97473500	-0.33635000
	C	5.14788200	-0.92815000	-0.35920900
	C	3.01197200	-0.01649100	-1.04534300
	C	5.80417400	0.06016500	-1.07951500
	H	5.69965900	-1.67779700	0.19401900
	C	3.67448100	0.97408900	-1.76622200
	H	1.92869900	-0.02876700	-1.03002300
	C	5.06717200	1.01380200	-1.78564900

	H	6.88765800	0.09096700	-1.09415900
	H	3.10113800	1.71677600	-2.30884500
	H	5.57959300	1.78614600	-2.34856500
	O	3.73178200	-2.91686200	1.02723100
	H	-0.67645332	3.20863782	-0.44508441
	C	-2.51143635	3.37000884	-1.77590997
	C	-3.67180085	3.72149926	-1.08630367
	C	-2.38727666	3.67261142	-3.13216434
	C	-4.70838717	4.37540272	-1.75291488
	H	-3.76959894	3.48332621	-0.01726112
	C	-3.42376753	4.32593323	-3.79879341
	H	-1.47209531	3.39572154	-3.67553282
	C	-4.58482774	4.67710569	-3.10898645
	H	-5.62338292	4.65168998	-1.20914111
	H	-3.32654867	4.56397131	-4.86799064
	H	-5.40209159	5.19195781	-3.63463234
**TS3a**	C	-5.55523800	-2.40280400	0.43445200
	C	-4.35139100	-2.89160300	0.92990800
	C	-3.14693200	-2.31099100	0.53398800
	C	-3.15098500	-1.23007300	-0.36101000
	C	-4.36373200	-0.73750700	-0.84980000
	C	-5.56013000	-1.32830500	-0.45227300
	H	-1.94368700	-3.55444600	1.68268000
	H	-6.48625200	-2.86280600	0.74287100
	H	-4.33651200	-3.72618900	1.62210900
	H	-4.35677900	0.08388500	-1.54890400
	H	-6.49564800	-0.94487600	-0.84062500
	C	-0.70511000	-0.94996500	0.01040200
	C	-0.71397900	-2.15190300	0.93889500
	C	-1.62617700	0.01772000	-1.89568800
	C	-0.08847100	0.16431100	-1.95015100
	C	0.45801400	-0.50162100	-0.75689200
	N	-1.89262300	-0.72310800	-0.75215000
	N	-1.92532600	-2.78928400	1.02017000
	O	0.23395300	-2.48076400	1.61716500
	O	0.44924300	0.70617600	-2.89208500
	O	-2.41221300	0.43458600	-2.70802500
	C	0.93430500	1.31713300	0.58369400
	O	-0.85562000	0.21472500	1.40426300
	N	-0.36244600	1.37541100	0.96943400
	C	-1.27735400	2.40708600	0.58963900
	C	-0.80841300	3.66125500	0.17865800
	C	-2.65137900	2.18975500	0.73069900
	C	-1.71699600	4.66267900	-0.14805100
	H	0.25068000	3.87596500	0.13238600
	C	-3.54549500	3.20606100	0.41432000
	H	-3.00452200	1.23452700	1.08860600
	C	-3.08783700	4.44260900	-0.03665400
	H	-1.34405000	5.62572500	-0.47615700
	H	-4.60845200	3.02479100	0.52217600

	H	-3.78963500	5.22949300	-0.28503300
	C	1.62843900	-1.44829600	-0.95222000
	C	3.00672000	-0.96525700	-1.29427000
	C	4.06345500	-1.84487700	-1.01181600
	C	3.28974200	0.25787800	-1.91228100
	C	5.37262200	-1.50112800	-1.32168000
	H	3.83478000	-2.79446700	-0.54562300
	C	4.60100600	0.58671100	-2.24994800
	H	2.48420200	0.92587100	-2.18142600
	C	5.64518100	-0.28393300	-1.94781100
	H	6.18039400	-2.18372300	-1.08346000
	H	4.80473600	1.52531000	-2.75314800
	H	6.66539400	-0.02011100	-2.20364900
	O	1.42553500	-2.64576600	-0.86277100
	H	1.57418854	0.55258210	0.97216662
	C	1.48806129	2.35123890	-0.41409303
	C	2.21412989	3.44836092	0.04925975
	C	1.26388020	2.19031755	-1.78166644
	C	2.71590606	4.38496582	-0.85487706
	H	2.39114880	3.57508749	1.12710367
	C	1.76509999	3.12690977	-2.68560823
	H	0.69172333	1.32496733	-2.14674142
	C	2.49103906	4.22476460	-2.22198793
	H	3.28762729	5.25021562	-0.48920042
	H	1.58797750	3.00077615	-3.76357589
	H	2.88597660	4.96329955	-2.93464983
**5a**	C	-5.60412000	-2.34720000	0.71987200
	C	-4.39897700	-2.74154700	1.28999900
	C	-3.20473900	-2.17069800	0.85193100
	C	-3.22176600	-1.19642500	-0.15591100
	C	-4.43567800	-0.79730900	-0.71931000
	C	-5.62213100	-1.37683400	-0.28072400
	H	-1.96018500	-3.33816500	2.04581700
	H	-6.52838200	-2.79816800	1.06016300
	H	-4.37580200	-3.49260200	2.07172000
	H	-4.43790600	-0.05727000	-1.50460600
	H	-6.56051500	-1.06698900	-0.72362900
	C	-0.84582700	-0.63986800	0.37687800
	C	-0.78396400	-1.91693500	1.25123600
	C	-1.69444600	-0.18300100	-1.81787100
	C	-0.22155600	0.21991300	-1.79487400
	C	0.40771100	-0.24764600	-0.46051900
	N	-1.96961300	-0.68129500	-0.56424600
	N	-1.97606400	-2.56128500	1.39701800
	O	0.22117800	-2.25559300	1.83975500
	O	0.32771400	0.81473300	-2.67841600
	O	-2.43957700	-0.05824700	-2.75916400
	C	0.98657500	1.01753800	0.27190600
	O	-0.97879000	0.40156000	1.36033000
	N	-0.20701400	1.54068500	0.97387400

	C	-0.98413700	2.59002400	0.40849100
	C	-0.33145900	3.71284300	-0.12448800
	C	-2.38281300	2.58815100	0.48578600
	C	-1.07153600	4.78502100	-0.61079900
	H	0.74977000	3.76360500	-0.13951800
	C	-3.10805800	3.67297800	-0.00031300
	H	-2.89352600	1.74691200	0.93094400
	C	-2.46404800	4.77395600	-0.55832300
	H	-0.54884600	5.63926500	-1.02603600
	H	-4.19031100	3.64995800	0.06171400
	H	-3.03508000	5.61313900	-0.93667000
	C	1.26136900	-1.49059900	-0.85095200
	C	2.70477400	-1.38000800	-1.19258200
	C	3.50626100	-2.50136900	-0.93142500
	C	3.26062000	-0.26787000	-1.83475800
	C	4.84975000	-2.49630000	-1.28127400
	H	3.06133600	-3.36098800	-0.44682000
	C	4.59971300	-0.28208200	-2.21407600
	H	2.64242000	0.58291700	-2.08726900
	C	5.39765200	-1.38762900	-1.92817500
	H	5.46888800	-3.35717100	-1.05780800
	H	5.01918100	0.57191400	-2.73282400
	H	6.44386100	-1.38838900	-2.21218400
	O	0.67918800	-2.55244900	-0.93524500
	H	1.74777835	0.73868041	0.97026687
	C	1.51898901	2.04433727	-0.74486270
	C	0.76482451	2.36354406	-1.87397344
	C	2.75556945	2.65569373	-0.53624347
	C	1.24725290	3.29396677	-2.79486857
	H	-0.20997474	1.88197829	-2.03830137
	C	3.23816111	3.58555017	-1.45711767
	H	3.34978873	2.40412190	0.35431214
	C	2.48388322	3.90449403	-2.58696555
	H	0.65279244	3.54483500	-3.68533119
	H	4.21314478	4.06710157	-1.29337575
	H	2.86448869	4.63740654	-3.31310275
**MC4a**	C	5.48106000	0.01625500	-1.26313600
	C	4.88359100	1.06994300	-0.58232900
	C	3.50941000	1.28230600	-0.68833700
	C	2.72683200	0.43287300	-1.48824500
	C	3.33230200	-0.62715000	-2.16801000
	C	4.70415200	-0.82943300	-2.05205900
	H	3.46433900	2.88959400	0.63350500
	H	6.54890800	-0.14189600	-1.17527200
	H	5.47480800	1.73397000	0.03828000
	H	2.72650900	-1.27704000	-2.77972800
	H	5.16322900	-1.65303700	-2.58491600
	C	0.74183100	1.80152400	-0.90691900
	C	1.56097600	2.63056900	0.01968600
	C	0.36635100	0.03345000	-2.29997400

	C	-0.95469500	0.83016700	-2.05060200
	C	-0.58890700	1.92733200	-1.14928800
	N	1.34760600	0.71585700	-1.57149800
	N	2.89614800	2.33107000	0.00903600
	O	1.07582500	3.48553800	0.73657700
	O	-2.00393400	0.54386400	-2.57062100
	O	0.50727600	-0.93249400	-3.00252000
	N	-0.62850500	-2.25228200	0.55583800
	C	-1.44271400	-3.26933600	-0.10278800
	C	-1.70231500	-3.12515100	-1.46373300
	C	-1.98201300	-4.32903900	0.62581800
	C	-2.48287300	-4.08116200	-2.10576900
	H	-1.29823900	-2.28253200	-2.00608400
	C	-2.76532300	-5.27604300	-0.02926500
	H	-1.82502100	-4.39962400	1.69465600
	C	-3.01230700	-5.15794100	-1.39522600
	H	-2.67960200	-3.97884400	-3.16632200
	H	-3.19487700	-6.09533300	0.53506800
	H	-3.62565200	-5.89425800	-1.90129300
	C	-1.49380100	3.06807100	-0.76752100
	C	-2.67379000	2.78773700	0.09437700
	C	-3.55827100	3.84255700	0.36603700
	C	-2.91751500	1.52218800	0.64301200
	C	-4.66829500	3.63582000	1.17219400
	H	-3.35178800	4.81365500	-0.06611900
	C	-4.03370000	1.31998400	1.45185100
	H	-2.24823800	0.69222800	0.44661000
	C	-4.90778500	2.37164700	1.71698200
	H	-5.34882700	4.45402900	1.37837500
	H	-4.21947800	0.33885700	1.87341800
	H	-5.77588400	2.20931200	2.34633100
	O	-1.26342400	4.17683900	-1.21251400
	O	-0.79252000	-1.04042400	0.14757200
	C	0.21106300	-2.60891500	1.50581100
	H	0.74374994	-1.86236711	2.05700142
	C	0.42611327	-4.10040284	1.82332854
	C	1.24141485	-4.87814765	1.00117011
	C	-0.19423414	-4.67353092	2.93378034
	C	1.43628195	-6.22928501	1.28913898
	H	1.73065589	-4.42641758	0.12611241
	C	0.00016056	-6.02444954	3.22147219
	H	-0.83667994	-4.05997057	3.58207141
	C	0.81540782	-6.80262405	2.39865212
	H	2.07849338	-6.84235522	0.64033148
	H	-0.48922495	-6.47671307	4.09626123
	H	0.96845464	-7.86774679	2.62533854
**TS4a**	C	-5.03542800	-2.17539200	-1.09602800
	C	-3.78426500	-2.76619300	-1.24649900
	C	-2.63788700	-1.97251400	-1.28106300
	C	-2.75067500	-0.57841800	-1.14571800

C	-4.00731200	0.00833400	-0.98495900
C	-5.14634100	-0.79261400	-0.96703800
H	-1.30436900	-3.53333000	-1.64761500
H	-5.92129700	-2.79882900	-1.08120400
H	-3.69085600	-3.84161500	-1.35033300
H	-4.08043100	1.08195000	-0.89972300
H	-6.11926500	-0.33125900	-0.85075900
C	-0.27455900	-0.46477300	-0.88931500
C	-0.15931600	-1.92130700	-1.24296600
C	-1.40892900	1.46179600	-1.65069100
C	0.11524800	1.71829100	-1.66589300
C	0.75723000	0.48982900	-1.15367300
N	-1.55298400	0.16200600	-1.20726500
N	-1.36882700	-2.54092400	-1.46207100
O	0.89140800	-2.52389800	-1.27429100
O	0.62616300	2.72571000	-2.09583900
O	-2.27397000	2.23724600	-1.98182800
N	-0.17876700	0.73243700	1.35907700
C	-1.25699100	1.61027400	1.70705900
C	-1.18482600	2.95839600	1.34258900
C	-2.32795800	1.14253600	2.47793400
C	-2.21125700	3.82050600	1.70922300
H	-0.33729300	3.31578600	0.77622800
C	-3.34618100	2.01885800	2.83739600
H	-2.35796100	0.11762800	2.82300400
C	-3.29694500	3.35710200	2.45099900
H	-2.16000000	4.86041800	1.41012100
H	-4.17393100	1.65322500	3.43327900
H	-4.09198200	4.03568400	2.73581800
C	2.14096600	0.15867600	-1.69133900
C	3.38775200	0.64364900	-1.03811400
C	4.55122000	-0.11582000	-1.23417600
C	3.46143000	1.84723600	-0.32718500
C	5.76157300	0.30625600	-0.70016700
H	4.48341600	-1.03538600	-1.80181700
C	4.68308800	2.27977400	0.18272000
H	2.57838000	2.45484400	-0.19609200
C	5.82975600	1.50789600	0.00767100
H	6.65318400	-0.29394400	-0.84070100
H	4.73809000	3.22120200	0.71705400
H	6.77634000	1.84309800	0.41674600
O	2.18027200	-0.46813000	-2.73012300
O	0.88506200	1.23455000	0.83491100
C	-0.34114000	-0.62375700	1.14812300
H	0.49362688	-1.22229279	0.84842455
C	-1.72350712	-1.27347565	1.34440554
C	-2.65708134	-1.25364036	0.30826179
C	-2.04181394	-1.88243868	2.55859444
C	-3.90929284	-1.84251036	0.48622214
H	-2.40615442	-0.77398241	-0.64887287

	C	-3.29387741	-2.47077148	2.73674778
	H	-1.30538201	-1.89828572	3.37522845
	C	-4.22801756	-2.45046356	1.70031818
	H	-4.64536307	-1.82602950	-0.33058309
	H	-3.54538521	-2.95023077	3.69390970
	H	-5.21523197	-2.91408726	1.84087878
6a	C	4.95960300	2.73453300	-0.72885800
	C	4.14284200	2.54201300	0.37973300
	C	2.85998200	2.02029200	0.22106900
	C	2.39847100	1.67574500	-1.06079200
	C	3.22568100	1.86818100	-2.17108000
	C	4.50078500	2.39877400	-2.00084300
	H	2.31443000	2.21241400	2.22376800
	H	5.95242500	3.14690700	-0.59599400
	H	4.49139900	2.79961600	1.37351300
	H	2.85556100	1.61974200	-3.15354200
	H	5.13322100	2.54827900	-2.86717000
	C	0.45082500	0.51817000	0.00663400
	C	0.83423100	1.17520400	1.33680200
	C	0.29147200	1.16037600	-2.28385700
	C	-1.06899700	0.60528000	-1.83923500
	C	-1.06310400	0.56128200	-0.29238900
	N	1.08528000	1.16206900	-1.15536700
	N	2.01873300	1.84709000	1.32734400
	O	0.14833200	1.03912700	2.32945300
	O	-1.95555000	0.26749000	-2.56981200
	O	0.57582500	1.51648800	-3.40424900
	N	-0.61434600	-1.62071100	0.35753100
	C	-1.02190600	-2.80769200	-0.30121000
	C	-2.38414900	-3.09228000	-0.47524500
	C	-0.06818400	-3.75516000	-0.70299500
	C	-2.77456100	-4.29063400	-1.06182400
	H	-3.12423900	-2.37475400	-0.15346400
	C	-0.47887300	-4.95488900	-1.27967600
	H	0.98747700	-3.57728000	-0.54999200
	C	-1.82956600	-5.23111500	-1.46910900
	H	-3.83179600	-4.48900200	-1.19822700
	H	0.27262000	-5.67491000	-1.58366700
	H	-2.14182500	-6.16417500	-1.92264700
	C	-1.75521100	1.86220400	0.21483200
	C	-3.19821000	1.84317800	0.55516500
	C	-3.67410400	2.87323700	1.38145500
	C	-4.09443900	0.89516800	0.04266300
	C	-5.02087700	2.93963200	1.70870500
	H	-2.97157600	3.60449600	1.76046600
	C	-5.44744400	0.98164000	0.35566900
	H	-3.74361100	0.11517600	-0.61878900
	C	-5.91009700	1.99412900	1.19403100
	H	-5.38095200	3.72721400	2.36016300
	H	-6.14041300	0.25676800	-0.05481200

	H	-6.96334600	2.04932100	1.44498900
	O	-1.08147000	2.87055600	0.26706800
	O	-1.63438800	-0.59230500	0.27087200
	C	0.70047500	-1.04395100	0.03193200
	H	1.41610420	-1.29574661	0.78650063
	C	1.17615116	-1.53486875	-1.34804334
	C	0.28363802	-1.57720735	-2.41910630
	C	2.50010789	-1.93678114	-1.52697047
	C	0.71484634	-2.02177427	-3.66926272
	H	-0.75980079	-1.26013376	-2.27824246
	C	2.93116367	-2.38170132	-2.77668002
	H	3.20369513	-1.90290209	-0.68240465
	C	2.03811566	-2.42453086	-3.84805644
	H	0.01076546	-2.05576184	-4.51327500
	H	3.97448080	-2.69925542	-2.91790711
	H	2.37791249	-2.77554865	-4.83325750
MC1b	C	-5.49334900	-2.41065900	0.46166500
	C	-4.27219100	-2.88476100	0.92916600
	C	-3.09970100	-2.28131600	0.49959700
	C	-3.12591500	-1.20384100	-0.38836400
	C	-4.35544000	-0.72539100	-0.85083800
	C	-5.53077100	-1.33510700	-0.42465800
	H	-6.41175300	-2.88120700	0.79038800
	H	-4.20456200	-3.71620800	1.61909700
	H	-4.37536900	0.09627800	-1.54981200
	H	-6.48042100	-0.96576200	-0.79193800
	C	-0.67637600	-1.08952700	-0.20506900
	C	-0.71117700	-2.15516400	0.84563900
	C	-1.62401000	0.10409800	-1.91610700
	C	-0.07785800	0.22614500	-1.97510200
	C	0.43322700	-0.60931500	-0.89357300
	N	-1.88598300	-0.68701400	-0.79829300
	O	0.19985600	-2.48579200	1.54166300
	O	0.46990300	0.86016400	-2.85072900
	O	-2.41348800	0.55954800	-2.69929000
	C	0.95038900	1.46025400	0.77284500
	O	-0.85156300	0.32302700	1.57561400
	H	1.23697500	2.24942700	0.09499700
	N	-0.34954600	1.37792700	1.01737500
	C	2.01661000	0.86172600	1.54144000
	C	3.30149000	1.39209800	1.29977400
	C	1.84101900	-0.03294000	2.61449600
	C	4.36712700	1.07750600	2.12636200
	H	3.44818600	2.07929800	0.47550500
	C	2.91322100	-0.32965500	3.44542500
	H	0.87338900	-0.47127700	2.79082200
	C	4.17217700	0.22346700	3.21202500
	H	5.34428200	1.50348600	1.93290500
	H	2.76531000	-1.00464100	4.28014400
	H	5.00069000	-0.01700100	3.86834300

	C	-1.26387700	2.43550300	0.63355700
	C	-0.79061200	3.68610400	0.22732100
	C	-2.63264400	2.20847400	0.77304300
	C	-1.69636600	4.68802300	-0.09490600
	H	0.26555200	3.90831900	0.18384500
	C	-3.52762400	3.22741900	0.46334800
	H	-2.97503900	1.24986500	1.13217400
	C	-3.06662400	4.46445600	0.01905100
	H	-1.32457300	5.65334600	-0.41660100
	H	-4.59077900	3.04935600	0.57198100
	H	-3.76814800	5.25361900	-0.22310300
	C	1.67170600	-1.44138700	-1.00310700
	C	3.03515400	-0.90703600	-1.30834900
	C	4.09804300	-1.78758700	-1.05543700
	C	3.30801200	0.34124900	-1.88006300
	C	5.40288200	-1.42952600	-1.35857000
	H	3.87258800	-2.75422200	-0.62508100
	C	4.61795700	0.68763800	-2.20814800
	H	2.49923300	1.01538500	-2.12241100
	C	5.66647800	-0.19034000	-1.94418400
	H	6.21419200	-2.11717300	-1.14899800
	H	4.81713800	1.64463600	-2.67732500
	H	6.68392800	0.08555200	-2.19824600
	O	1.51616300	-2.64607100	-0.89511600
	O	-1.91354800	-2.79357300	0.98957200
**TS1b**	C	-5.57133900	-2.34679700	0.43493000
	C	-4.37158200	-2.86159500	0.91252800
	C	-3.15969700	-2.30520200	0.50397300
	C	-3.15417800	-1.21885800	-0.38601300
	C	-4.36308200	-0.70075200	-0.85792600
	C	-5.56552400	-1.26952600	-0.44783400
	H	-6.50759300	-2.78938700	0.75324000
	H	-4.36514900	-3.69959500	1.60113700
	H	-4.34670300	0.12366700	-1.55337400
	H	-6.49793600	-0.86665200	-0.82394500
	C	-0.70319400	-1.00788900	-0.05799600
	C	-0.71365100	-2.20297000	0.85973900
	C	-1.63361400	0.06818800	-1.89725600
	C	-0.09502200	0.21505000	-1.94827800
	C	0.45419300	-0.49765100	-0.77134600
	N	-1.89225800	-0.72967300	-0.78478900
	O	0.23791700	-2.56597700	1.51268000
	O	0.45001400	0.80308000	-2.85275700
	O	-2.42573900	0.53283400	-2.67363700
	C	0.91495200	1.24472000	0.54574100
	O	-0.84591000	0.18378400	1.45975700
	H	1.17939200	2.02494800	-0.15774800
	N	-0.36872000	1.32332000	0.99027000
	C	2.02782300	0.82364600	1.41468100
	C	3.26152600	1.46345500	1.20128500

	C	1.91354600	-0.08451300	2.47972000
	C	4.34849400	1.21315500	2.02868100
	H	3.36562100	2.16696500	0.38371000
	C	3.00515200	-0.32785100	3.30474000
	H	0.98485500	-0.60521100	2.64368300
	C	4.22298200	0.31399100	3.08503500
	H	5.29017800	1.71756700	1.84670000
	H	2.90381700	-1.03413700	4.12060500
	H	5.07016700	0.11092000	3.73044300
	C	-1.29140300	2.35612900	0.63008200
	C	-0.84390000	3.59140600	0.14551000
	C	-2.65543700	2.15554300	0.86605400
	C	-1.76539800	4.58965800	-0.15290200
	H	0.21083000	3.79429200	0.01787500
	C	-3.56155000	3.16796300	0.57485700
	H	-2.98569000	1.21311700	1.27677300
	C	-3.12690500	4.38578800	0.05565200
	H	-1.40911300	5.53769000	-0.53863400
	H	-4.61656700	2.99911100	0.75700900
	H	-3.83829400	5.17034200	-0.17232900
	C	1.63842300	-1.43296200	-1.00206000
	C	3.02438000	-0.93155500	-1.28055000
	C	4.07636400	-1.78776800	-0.92240500
	C	3.31758100	0.27437000	-1.92612200
	C	5.39383700	-1.43288200	-1.17982700
	H	3.83695500	-2.72774100	-0.44200700
	C	4.63902700	0.61053000	-2.21315100
	H	2.51513400	0.91879600	-2.25752600
	C	5.67920800	-0.23290900	-1.83187000
	H	6.19867800	-2.09540100	-0.88200800
	H	4.85339900	1.53298000	-2.74152500
	H	6.70684100	0.03766800	-2.04851400
	O	1.43231100	-2.62969200	-0.98949000
	O	-1.94299000	-2.81310700	0.96590100
	C	-5.48265100	-2.40678700	0.46069600
	C	-4.26146700	-2.88308800	0.92826500
	C	-3.09002000	-2.28198400	0.49172700
	C	-3.11077600	-1.20431200	-0.39455900
	C	-4.34183100	-0.72614300	-0.85789000
	C	-5.51858000	-1.33401400	-0.42937500
	H	-6.40171400	-2.87470500	0.79145400
	H	-4.19506100	-3.71536000	1.61721100
**3b**	H	-4.36115400	0.09908800	-1.55272000
	H	-6.46797800	-0.96259300	-0.79504200
	C	-0.72753800	-0.79508200	0.14109300
	C	-0.71414000	-2.13425100	0.90539300
	C	-1.59697500	0.08513900	-1.89183700
	C	-0.07392700	0.29191300	-1.91171900
	C	0.53460900	-0.26660500	-0.60540500
	N	-1.85459700	-0.68078500	-0.77565300

	O	0.21363600	-2.51902500	1.54420900
	O	0.47682900	0.83036800	-2.83341700
	O	-2.37886400	0.51420000	-2.70424500
	C	0.93512500	1.08451500	0.44138200
	O	-0.90771900	0.10932800	1.31211500
	H	1.23849100	1.91000500	-0.19931800
	N	-0.34731300	1.36714200	1.03573000
	C	2.02654700	0.80428700	1.47226000
	C	3.28808500	1.37967700	1.27677700
	C	1.83966600	-0.02229200	2.59147900
	C	4.35139600	1.09532700	2.13124200
	H	3.45093200	2.04174800	0.43454300
	C	2.90304500	-0.30528700	3.44565100
	H	0.87539500	-0.46556500	2.78384600
	C	4.16504500	0.23881700	3.21258600
	H	5.32214000	1.54054800	1.94607000
	H	2.74271400	-0.96260100	4.29261100
	H	4.99245300	0.00293000	3.87219800
	C	-1.25593100	2.38850100	0.63499700
	C	-0.77831400	3.64450600	0.22666000
	C	-2.63836300	2.20161200	0.77702600
	C	-1.67597700	4.66319100	-0.08712500
	H	0.28560200	3.83832500	0.16951900
	C	-3.51986800	3.23158700	0.46433100
	H	-3.01510300	1.25579600	1.13714600
	C	-3.05071200	4.46585500	0.02089800
	H	-1.28790800	5.62215900	-0.41125700
	H	-4.58491200	3.06045700	0.57309700
	H	-3.74193900	5.26310400	-0.22389200
	C	1.65057700	-1.34429800	-0.94366300
	C	3.02767100	-0.88621000	-1.29959500
	C	4.07780300	-1.78631700	-1.05537100
	C	3.31495400	0.35338600	-1.88172500
	C	5.38716500	-1.43785700	-1.35999800
	H	3.84960000	-2.74761300	-0.61340800
	C	4.62459000	0.68552400	-2.21471100
	H	2.51875000	1.04168600	-2.12156700
	C	5.66385400	-0.20075200	-1.94402800
	H	6.19237000	-2.13113900	-1.14675000
	H	4.83123600	1.63983700	-2.68520300
	H	6.68495700	0.06730200	-2.19073700
	O	1.39018000	-2.52802200	-0.92032200
	O	-1.89517200	-2.81032300	0.95646300
**MC2b**	C	5.01479900	2.24454100	-0.98319100
	C	3.74800600	2.80704900	-1.10933500
	C	2.63789400	1.97802000	-1.18485100
	C	2.77222800	0.58702000	-1.13244900
	C	4.04583500	0.02663400	-0.99173300
	C	5.15911700	0.85912400	-0.92413700
	H	5.88425800	2.88848900	-0.93422500

H	3.59955300	3.87810500	-1.16478500
H	4.14959300	-1.04768000	-0.96409800
H	6.14369900	0.41831900	-0.82692500
C	0.30933900	0.45146100	-1.12286400
C	0.21525500	1.91773500	-1.20627300
C	1.45384500	-1.48823100	-1.63292700
C	-0.08748300	-1.72877200	-1.65609300
C	-0.71284400	-0.44906500	-1.26747600
N	1.59434500	-0.17029800	-1.23783100
O	-0.80467900	2.54366100	-1.19113600
O	-0.60179800	-2.74725700	-2.04364300
O	2.31808600	-2.27409800	-1.92955000
N	0.15680200	-0.72280900	1.41243400
C	1.19399100	-1.67121000	1.75680600
C	1.06170700	-2.99323100	1.32954300
C	2.25752800	-1.28836400	2.57620400
C	2.03198900	-3.92182000	1.69008200
H	0.20987500	-3.28148700	0.73225800
C	3.22284400	-2.22483800	2.92133300
H	2.32355800	-0.28829500	2.98036300
C	3.11698200	-3.54124900	2.47711000
H	1.93696500	-4.94630000	1.35162600
H	4.04781400	-1.92679900	3.55704700
H	3.86869300	-4.26985300	2.75601500
C	-2.11632900	-0.12198100	-1.71202400
C	-3.35628900	-0.60245000	-1.03024200
C	-4.50292500	0.18809300	-1.20760600
C	-3.46368300	-1.81900700	-0.34464500
C	-5.72732900	-0.21683500	-0.69350700
H	-4.41161600	1.11778100	-1.75455700
C	-4.70123800	-2.23554700	0.14415100
H	-2.59491300	-2.44808700	-0.21696100
C	-5.83043300	-1.43567900	-0.02103000
H	-6.60231300	0.40929700	-0.82533400
H	-4.78162700	-3.18840900	0.65462400
H	-6.78825300	-1.76162200	0.36903100
O	-2.19119500	0.53327400	-2.73155400
O	-0.93604000	-1.20293000	0.94446400
H	1.38892700	0.87422700	1.67119100
C	0.36084600	0.59090600	1.49961200
C	-0.63766800	1.60676000	1.74460200
C	-0.14886800	2.90148900	2.01180900
C	-2.01459300	1.35517100	1.88275600
C	-1.00006700	3.90229800	2.45238200
H	0.91017000	3.10713400	1.89877900
C	-2.85461300	2.35968400	2.34451400
H	-2.40361100	0.37534800	1.65130300
C	-2.35538500	3.62917100	2.63571300
H	-0.60811100	4.89017500	2.66349000
H	-3.91042100	2.15144600	2.47224200

	H	-3.02158600	4.40548100	2.99379700
	O	1.40496600	2.59316200	-1.33958100
**TS2b**	C	-5.03542800	-2.17539200	-1.09602800
	C	-3.78426500	-2.76619300	-1.24649900
	C	-2.63788700	-1.97251400	-1.28106300
	C	-2.75067500	-0.57841800	-1.14571800
	C	-4.00731200	0.00833400	-0.98495900
	C	-5.14634100	-0.79261400	-0.96703800
	H	-5.92129700	-2.79882900	-1.08120400
	H	-3.69085600	-3.84161500	-1.35033300
	H	-4.08043100	1.08195000	-0.89972300
	H	-6.11926500	-0.33125900	-0.85075900
	C	-0.27455900	-0.46477300	-0.88931500
	C	-0.15931600	-1.92130700	-1.24296600
	C	-1.40892900	1.46179600	-1.65069100
	C	0.11524800	1.71829100	-1.66589300
	C	0.75723000	0.48982900	-1.15367300
	N	-1.55298400	0.16200600	-1.20726500
	O	0.89140800	-2.52389800	-1.27429100
	O	0.62616300	2.72571000	-2.09583900
	O	-2.27397000	2.23724600	-1.98182800
	N	-0.17876700	0.73243700	1.35907700
	C	-1.25699100	1.61027400	1.70705900
	C	-1.18482600	2.95839600	1.34258900
	C	-2.32795800	1.14253600	2.47793400
	C	-2.21125700	3.82050600	1.70922300
	H	-0.33729300	3.31578600	0.77622800
	C	-3.34618100	2.01885800	2.83739600
	H	-2.35796100	0.11762800	2.82300400
	C	-3.29694500	3.35710200	2.45099900
	H	-2.16000000	4.86041800	1.41012100
	H	-4.17393100	1.65322500	3.43327900
	H	-4.09198200	4.03568400	2.73581800
	C	2.14096600	0.15867600	-1.69133900
	C	3.38775200	0.64364900	-1.03811400
	C	4.55122000	-0.11582000	-1.23417600
	C	3.46143000	1.84723600	-0.32718500
	C	5.76157300	0.30625600	-0.70016700
	H	4.48341600	-1.03538600	-1.80181700
	C	4.68308800	2.27977400	0.18272000
	H	2.57838000	2.45484400	-0.19609200
	C	5.82975600	1.50789600	0.00767100
	H	6.65318400	-0.29394400	-0.84070100
	H	4.73809000	3.22120200	0.71705400
	H	6.77634000	1.84309800	0.41674600
	O	2.18027200	-0.46813000	-2.73012300
	O	0.88506200	1.23455000	0.83491100
	H	-1.36186500	-0.94735200	1.32160600
	C	-0.34114000	-0.62375700	1.14812300
	C	0.66359500	-1.60015000	1.63115200

	C	0.19147900	-2.87795900	1.97062600
	C	2.02470500	-1.31365800	1.81101100
	C	1.05236700	-3.84557400	2.47611100
	H	-0.86115400	-3.11092400	1.84780900
	C	2.87884500	-2.28172900	2.32765700
	H	2.40826200	-0.33885600	1.54912100
	C	2.40127400	-3.54940900	2.65736400
	H	0.66857600	-4.82596500	2.73374300
	H	3.92764800	-2.04509300	2.46442500
	H	3.07596700	-4.30058500	3.05159000
	O	-1.36882700	-2.54092400	-1.46207100
	C	5.00909300	2.24491100	-0.98710300
	C	3.74283500	2.80897600	-1.11550800
	C	2.63620400	1.97492200	-1.18798900
	C	2.77223600	0.58683200	-1.10781000
	C	4.04651800	0.02436000	-0.98487400
	C	5.15809600	0.85908600	-0.93036900
	H	5.87805300	2.88961200	-0.93810300
	H	3.59613500	3.87967000	-1.17926800
	H	4.14870500	-1.04935900	-0.94051300
	H	6.14452300	0.42204200	-0.83635200
	C	0.34391700	0.49888200	-0.63476900
	C	0.20832200	1.92108600	-1.15977800
	C	1.43643200	-1.44772400	-1.61054300
	C	-0.06797100	-1.71787700	-1.61076100
	C	-0.74716000	-0.56008200	-0.87047900
	N	1.57587700	-0.15306000	-1.14274200
	O	-0.81080000	2.53293000	-1.23814800
	O	-0.59996600	-2.69391100	-2.06214400
	O	2.29914300	-2.21638700	-1.96198000
**4b**	N	0.10601000	-0.73655700	1.40890700
	C	1.13883500	-1.67394500	1.71595700
	C	1.04285600	-3.01310600	1.32366700
	C	2.21209700	-1.27712600	2.53599300
	C	2.03034300	-3.92283400	1.69612500
	H	0.19942200	-3.34208300	0.73370600
	C	3.18804500	-2.20025400	2.90192500
	H	2.27468200	-0.26187100	2.90795900
	C	3.11067100	-3.52699100	2.47911400
	H	1.94450200	-4.95176200	1.36655200
	H	4.00949700	-1.87583800	3.53068800
	H	3.87272000	-4.24115400	2.76667700
	C	-2.08953300	-0.15956500	-1.58871900
	C	-3.37066000	-0.61479700	-1.00547600
	C	-4.50395300	0.18674100	-1.20408200
	C	-3.48883700	-1.83716400	-0.33440000
	C	-5.73295500	-0.21518400	-0.69833200
	H	-4.39946000	1.12112100	-1.74121700
	C	-4.72711500	-2.24552000	0.14630100
	H	-2.63014500	-2.47923500	-0.21041700

	C	-5.84513600	-1.43190100	-0.02265000
	H	-6.60511300	0.41356600	-0.83411300
	H	-4.81853300	-3.19901800	0.65290000
	H	-6.80665400	-1.74739400	0.36629000
	O	-2.03530400	0.45972000	-2.62846900
	O	-0.92869700	-1.16021200	0.55876700
	H	1.38377800	0.90109900	1.31589200
	C	0.36901500	0.62460400	1.03329100
	C	-0.61421600	1.62706400	1.64228100
	C	-0.13366400	2.89790600	1.97684200
	C	-1.97292000	1.35094900	1.86344500
	C	-0.98602600	3.88625500	2.46568600
	H	0.91824200	3.12886700	1.84109900
	C	-2.82364900	2.33883300	2.35711600
	H	-2.36963900	0.37033200	1.64167200
	C	-2.33879300	3.61336500	2.64642900
	H	-0.58965000	4.86697100	2.70213100
	H	-3.87254300	2.10990200	2.50727500
	H	-3.00711800	4.38259300	3.01613000
	O	1.39713200	2.57147600	-1.37757900
**MC3b**	C	-2.44083700	-4.83186900	-0.58946800
	C	-1.10498700	-4.69471300	-0.22640800
	C	-0.40927600	-3.55818200	-0.60888500
	C	-1.02262400	-2.54528600	-1.35310400
	C	-2.36548000	-2.68921900	-1.71614400
	C	-3.06356900	-3.82944500	-1.33140900
	H	-2.98896300	-5.71780900	-0.29405500
	H	-0.58599400	-5.45108200	0.34855500
	H	-2.84102000	-1.91003900	-2.29197200
	H	-4.10316500	-3.93166500	-1.61787400
	C	1.10686300	-1.33150200	-1.30085000
	C	1.72438700	-2.42948400	-0.52085300
	C	-0.60051900	-0.27166300	-2.38859000
	C	0.70205800	0.59625900	-2.41357100
	C	1.71453400	-0.18482500	-1.68465100
	N	-0.23677400	-1.43473200	-1.69923300
	O	2.86677900	-2.41265900	-0.15554400
	O	0.78426300	1.66863000	-2.95564800
	O	-1.65882100	-0.01531900	-2.89423200
	C	-2.02152600	1.35982000	1.98937700
	O	-0.61009900	0.87563100	0.22671500
	N	-1.63004600	1.48226600	0.73908500
	C	-2.35436400	2.38433800	-0.14917400
	C	-3.74678100	2.43628200	-0.13119100
	C	-1.61428200	3.16291000	-1.03451700
	C	-4.40346700	3.30722300	-0.99710600
	H	-4.31349200	1.78537300	0.52268600
	C	-2.28201000	4.03058400	-1.89172100
	H	-0.53730300	3.07874300	-1.05059600
	C	-3.67414200	4.10819200	-1.87319900

	H	-5.48640000	3.34433100	-0.99632500
	H	-1.71073400	4.63998900	-2.58137900
	H	-4.18914400	4.78002600	-2.54971300
	C	3.16805700	0.20305100	-1.57797000
	C	3.63578200	0.91991400	-0.36524200
	C	5.00419800	1.20333000	-0.24390000
	C	2.74984900	1.32939900	0.63925700
	C	5.48035200	1.87791900	0.87109300
	H	5.67280900	0.88169800	-1.03262700
	C	3.23250300	2.01161000	1.75373000
	H	1.68728100	1.13067300	0.55059200
	C	4.59384600	2.28322900	1.87229800
	H	6.53912700	2.09003800	0.96512400
	H	2.54488700	2.32899900	2.52902400
	H	4.96671500	2.81083800	2.74316300
	O	3.89327900	-0.04228900	-2.52238000
	O	0.92415000	-3.48240100	-0.21407100
	H	-1.52761558	0.66807893	2.63933607
	C	-3.19055239	2.21210650	2.51718852
	C	-4.46799399	1.66045086	2.61397258
	C	-2.97165830	3.53598688	2.89907334
	C	-5.52688145	2.43268962	3.09238251
	H	-4.64053286	0.61691024	2.31331307
	C	-4.03033962	4.30824513	3.37693111
	H	-1.96426152	3.97054987	2.82304069
	C	-5.30842178	3.75648203	3.47326406
	H	-6.53402875	1.99771574	3.16784001
	H	-3.85832192	5.35201260	3.67735701
	H	-6.14312626	4.36542205	3.84977036
TS3b	C	-5.50313500	-2.43338300	0.45050100
	C	-4.28168200	-2.90874600	0.91797500
	C	-3.10972500	-2.30688700	0.48463000
	C	-3.13371700	-1.22799100	-0.40107600
	C	-4.36378900	-0.75088100	-0.86543600
	C	-5.53993900	-1.35992900	-0.43834400
	H	-6.42179100	-2.90296400	0.78004800
	H	-4.21481300	-3.74140300	1.60650400
	H	-4.38262100	0.07133100	-1.56386100
	H	-6.48952000	-0.99038300	-0.80561500
	C	-0.71023700	-0.95473100	-0.03457100
	C	-0.72702900	-2.16816000	0.86546700
	C	-1.62310300	0.07014600	-1.91322100
	C	-0.08605500	0.23256900	-1.94632700
	C	0.45891800	-0.46928500	-0.76439100
	N	-1.88732600	-0.70695500	-0.79498700
	O	0.18951200	-2.52812300	1.53798200
	O	0.45855500	0.81563500	-2.85650600
	O	-2.41020700	0.50699900	-2.71287600
	C	0.92477900	1.27004100	0.61461900
	O	-0.87794200	0.18212400	1.41542100

	N	-0.37421000	1.34232400	1.00366400
	C	-1.27930500	2.38535600	0.62609600
	C	-0.79872200	3.63642600	0.22039900
	C	-2.65513100	2.17911100	0.76618100
	C	-1.69847400	4.64772500	-0.10093600
	H	0.26217900	3.84184400	0.17375100
	C	-3.54011100	3.20469200	0.45433600
	H	-3.01673300	1.22562000	1.12080400
	C	-3.07108100	4.43929300	0.00956700
	H	-1.31700400	5.60886400	-0.42464600
	H	-4.60464700	3.03278700	0.56162200
	H	-3.76578200	5.23365200	-0.23479800
	C	1.63454300	-1.41047200	-0.98394200
	C	3.01085800	-0.91909500	-1.31508900
	C	4.06628700	-1.81091900	-1.06662100
	C	3.29460500	0.32383800	-1.89227300
	C	5.37485400	-1.46000000	-1.36991400
	H	3.83742500	-2.77513000	-0.63172900
	C	4.60539700	0.66090800	-2.22291100
	H	2.49079000	1.00339900	-2.13574200
	C	5.64808400	-0.22252100	-1.95473400
	H	6.18167300	-2.15231600	-1.15848000
	H	4.80988700	1.61577200	-2.69397800
	H	6.66800900	0.04743300	-2.20500800
	O	1.42906800	-2.60867300	-0.91974600
	O	-1.91845000	-2.83027200	0.95816800
	H	1.55056365	0.48404751	0.98273286
	C	1.49728146	2.31930887	-0.35639999
	C	2.22564391	3.40191790	0.13654418
	C	1.28778823	2.18664044	-1.72932444
	C	2.74440367	4.35226183	-0.74334567
	H	2.39108593	3.50637473	1.21860728
	C	1.80598931	3.13697194	-2.60902593
	H	0.71381751	1.33272582	-2.11773359
	C	2.53422430	4.22030936	-2.11580147
	H	3.31793112	5.20606751	-0.35434195
	H	1.64044932	3.03311109	-3.69120233
	H	2.94255073	4.96967785	-2.80935242
	C	-5.49199200	-2.50961300	0.68263100
	C	-4.26414800	-2.84242100	1.24571500
	C	-3.11745100	-2.21521900	0.78256100
	C	-3.17324900	-1.25062000	-0.22409200
	C	-4.41020800	-0.91891900	-0.78595800
	C	-5.56093000	-1.55364500	-0.33057200
**5b**	H	-6.39176600	-2.99939700	1.03395900
	H	-4.17315200	-3.58421500	2.02876100
	H	-4.45349000	-0.18656700	-1.57750200
	H	-6.51633700	-1.29643700	-0.77092500
	C	-0.85120700	-0.61334800	0.34621100
	C	-0.76276900	-1.90376900	1.19294000

	C	-1.68398900	-0.08237600	-1.83054300
	C	-0.22545100	0.37254400	-1.76042900
	C	0.40656000	-0.17617900	-0.45619300
	N	-1.94943400	-0.66893800	-0.61309900
	O	0.22046800	-2.23992700	1.77958700
	O	0.30995100	1.04809400	-2.59205900
	O	-2.42575300	0.06906400	-2.76934600
	C	0.98683500	1.02915500	0.37050700
	O	-1.03267200	0.40370500	1.34819300
	N	-0.22455400	1.54726500	1.04227300
	C	-0.96419100	2.62199600	0.46907500
	C	-0.27125300	3.76078700	0.03004500
	C	-2.36387400	2.62239900	0.44650300
	C	-0.97188700	4.85463900	-0.46516600
	H	0.80871800	3.80431300	0.09481000
	C	-3.05029700	3.72982100	-0.04658600
	H	-2.90760500	1.76783500	0.82198200
	C	-2.36502600	4.84779000	-0.51282300
	H	-0.41945900	5.72220800	-0.80770000
	H	-4.13415000	3.71024400	-0.06319700
	H	-2.90553200	5.70402200	-0.89766800
	C	1.25320700	-1.40357200	-0.90940600
	C	2.68057700	-1.27658200	-1.30538100
	C	3.49715300	-2.39760800	-1.09301200
	C	3.20670500	-0.14966500	-1.94820900
	C	4.82712000	-2.37798200	-1.49057000
	H	3.07569100	-3.26836800	-0.60741100
	C	4.53113600	-0.15001100	-2.37627500
	H	2.57738200	0.70431500	-2.15960800
	C	5.34504900	-1.25545000	-2.13807500
	H	5.45853900	-3.23860300	-1.30393600
	H	4.92641300	0.71459400	-2.89650900
	H	6.38000100	-1.24549100	-2.46044300
	O	0.67636800	-2.46934000	-0.98549000
	O	-1.91166000	-2.60937000	1.34170800
	H	1.70523003	0.68982819	1.08721189
	C	1.59533568	2.09280887	-0.56221341
	C	0.90140016	2.50096000	-1.70125435
	C	2.84060026	2.64856942	-0.26749958
	C	1.45275466	3.46475378	-2.54599170
	H	-0.08023857	2.06321264	-1.93342989
	C	3.39209880	3.61179138	-1.11224204
	H	3.38732264	2.32686561	0.63087424
	C	2.69807360	4.01972565	-2.25203817
	H	0.90576495	3.78575857	-3.44432347
	H	4.37394664	4.04955115	-0.88064423
	H	3.13300432	4.77894624	-2.91815819
	C	5.41902500	0.82351000	-1.12137000
**MC4b**	C	4.69716900	1.73359300	-0.35633500
	C	3.31627500	1.77603100	-0.47323600

C	2.63477800	0.92471700	-1.34762800
C	3.36479700	0.00965300	-2.11209700
C	4.74990800	-0.03437800	-1.99285300
H	6.49773700	0.78586000	-1.03438400
H	5.18007700	2.41426800	0.33318700
H	2.84105500	-0.65006700	-2.78680800
H	5.30708400	-0.74614500	-2.58954300
C	0.54673300	2.01989100	-0.65462300
C	1.30017200	2.86115900	0.30942600
C	0.32115300	0.34120800	-2.18345500
C	-1.06386700	0.98590300	-1.85891100
C	-0.79112000	2.03454200	-0.86368400
N	1.23703000	1.05679700	-1.41479400
O	0.77506500	3.64494100	1.05891700
O	-2.08596200	0.66419300	-2.39094200
O	0.54291000	-0.52971600	-2.95674200
N	-0.52217000	-2.31722800	0.23550800
C	-1.60391300	-3.06303800	-0.39927400
C	-2.00822100	-2.69193400	-1.67907300
C	-2.23632300	-4.09707500	0.28869500
C	-3.04568300	-3.39310800	-2.28520000
H	-1.51644800	-1.87815200	-2.19244700
C	-3.27743700	-4.78682600	-0.32857100
H	-1.94204500	-4.34123500	1.30187700
C	-3.68027000	-4.43953200	-1.61596000
H	-3.35941100	-3.11584000	-3.28446300
H	-3.77991900	-5.58483100	0.20518900
H	-4.49252100	-4.97541000	-2.09295800
C	-1.80753000	3.02998500	-0.36371900
C	-2.83323900	2.57330100	0.60719500
C	-3.79546300	3.49962000	1.03820200
C	-2.86266400	1.26179000	1.09915000
C	-4.77032400	3.12126500	1.94944100
H	-3.75604000	4.50793900	0.64541200
C	-3.84572100	0.88757700	2.01245200
H	-2.13290600	0.52699900	0.77596000
C	-4.79635400	1.81245000	2.43834400
H	-5.51075600	3.83994800	2.28125300
H	-3.86841800	-0.12822900	2.38965500
H	-5.55903900	1.51642000	3.15008700
O	-1.77762000	4.16018500	-0.80894400
O	-0.57253500	-1.03054300	0.12798800
C	0.42139600	-2.97925000	0.87038600
O	2.65087400	2.69604400	0.33101900
H	1.17760265	-2.44785265	1.40952267
C	0.43725927	-4.51881532	0.83741667
C	0.92934952	-5.18752313	-0.28339785
C	-0.04015064	-5.24470717	1.92900757
C	0.94383662	-6.58229462	-0.31296200
H	1.30595536	-4.61546472	-1.14366528

	C	-0.02613695	-6.63914606	1.89932935
	H	-0.42770242	-4.71718952	2.81275130
	C	0.46569695	-7.30811374	0.77781332
	H	1.33114381	-7.10919333	-1.19705061
	H	-0.40302628	-7.21164164	2.75927097
	H	0.47650318	-8.40749003	0.75432890
**TS4b**	C	5.01272800	2.24228600	-1.02028800
	C	3.74540000	2.80452000	-1.14664500
	C	2.63654200	1.97293100	-1.21933200
	C	2.77392600	0.58281700	-1.14971900
	C	4.04879600	0.02279700	-1.02093500
	C	5.16095700	0.85686500	-0.96239400
	H	5.88153000	2.88742100	-0.97340600
	H	3.59759100	3.87527500	-1.20889000
	H	4.15164300	-1.05140200	-0.98692600
	H	6.14715500	0.41885800	-0.86930800
	C	0.32289500	0.46646100	-0.90282200
	C	0.21095900	1.91088200	-1.21520200
	C	1.44650800	-1.46816800	-1.65783900
	C	-0.07814200	-1.72084500	-1.66360200
	C	-0.72149300	-0.49095400	-1.11684000
	N	1.59012500	-0.16857600	-1.22011500
	O	-0.80695500	2.53673300	-1.24725000
	O	-0.60121400	-2.71799100	-2.09252800
	O	2.30913100	-2.24753600	-1.98468800
	N	0.13805500	-0.74184300	1.35459000
	C	1.16589300	-1.67851200	1.70209300
	C	1.05087000	-3.01041900	1.29301300
	C	2.23129300	-1.28183600	2.51934800
	C	2.03151900	-3.92551700	1.65922200
	H	0.20542900	-3.31952600	0.69640000
	C	3.20365800	-2.20979100	2.87616400
	H	2.29293700	-0.27131400	2.90081500
	C	3.11349200	-3.53181200	2.44412500
	H	1.94523500	-4.95264200	1.32587900
	H	4.02739700	-1.89680000	3.50666000
	H	3.87241100	-4.25094100	2.72770300
	C	-2.09649000	-0.14342300	-1.67935600
	C	-3.36055900	-0.61141900	-1.04936300
	C	-4.50045800	0.18462900	-1.23944200
	C	-3.47570700	-1.83153000	-0.37239100
	C	-5.72838200	-0.21840900	-0.73149400
	H	-4.40158900	1.11680600	-1.78133500
	C	-4.71498100	-2.24381300	0.11040100
	H	-2.61206300	-2.46797600	-0.24839400
	C	-5.83786500	-1.43609500	-0.05736800
	H	-6.60147800	0.40939900	-0.86685400
	H	-4.80249900	-3.19766000	0.61754100
	H	-6.79830500	-1.75620000	0.33080700
	O	-2.10454700	0.48980800	-2.71405000

	O	-0.92084400	-1.18065100	0.73758300
	C	0.35822200	0.60860100	1.21854500
	O	1.40200000	2.57799700	-1.39837700
	H	-0.44630041	1.25635509	0.93915580
	C	1.76271617	1.18879439	1.46828840
	C	2.71763858	1.16629511	0.45184111
	C	2.07973870	1.73744683	2.71122328
	C	3.98990432	1.69217950	0.67824102
	H	2.46774066	0.73417921	-0.52793933
	C	3.35182892	2.26279427	2.93779239
	H	1.32649130	1.75542134	3.51232844
	C	4.30730251	2.23980877	1.92105328
	H	4.74276680	1.67359460	-0.12306695
	H	3.60229779	2.69468514	3.91760459
	H	5.31029410	2.65376742	2.09977828
	C	5.12158300	2.40845700	0.27197900
	C	4.14396500	2.04913600	1.19427500
	C	2.86934700	1.73927000	0.74525300
	C	2.55389800	1.76525100	-0.61529500
	C	3.53983500	2.13051600	-1.53811600
	C	4.81591200	2.45236400	-1.08781700
	H	6.11722600	2.65815700	0.61743500
	H	4.34318600	2.01369300	2.25761700
	H	3.29125700	2.17297400	-2.58741300
	H	5.57392000	2.73765500	-1.80690100
	C	0.43971200	0.58917600	-0.06562700
	C	0.70790700	0.90773900	1.40734600
	C	0.57185100	1.81198700	-2.11576400
	C	-0.87363700	1.32562800	-1.94908800
	C	-1.03433900	0.78973700	-0.50382900
	N	1.23372500	1.42376600	-0.97093500
	O	-0.06098100	0.64478800	2.28401100
**6b**	O	-1.71489200	1.35545900	-2.79894300
	O	1.01150300	2.41289500	-3.06770300
	N	-0.70270700	-1.45239700	0.05871500
	C	-1.14050500	-2.74827100	-0.34242000
	C	-2.42021200	-3.14381300	0.06988500
	C	-0.34773500	-3.62994400	-1.08068700
	C	-2.90055500	-4.40210300	-0.26537900
	H	-3.02288200	-2.45340500	0.64604600
	C	-0.82962100	-4.90277600	-1.38801200
	H	0.64488400	-3.34814800	-1.40353300
	C	-2.10450200	-5.29118600	-0.99080200
	H	-3.89430600	-4.69667500	0.05191300
	H	-0.20232300	-5.58555000	-1.94950600
	H	-2.47631000	-6.27836500	-1.23910900
	C	-1.77101000	1.89403500	0.30598600
	C	-3.23057400	1.79908800	0.54391800
	C	-3.75821800	2.57170800	1.59067200
	C	-4.09231900	1.03721800	-0.25820300

C	-5.12206700	2.56434800	1.84590200
H	-3.08140600	3.16404600	2.19325900
C	-5.46165900	1.05278400	-0.01057000
H	-3.70355700	0.45599500	-1.08232100
C	-5.97625600	1.80551300	1.04336500
H	-5.52231900	3.15073800	2.66461500
H	-6.12670300	0.47489100	-0.64143600
H	-7.04271100	1.80419900	1.23866900
O	-1.10504800	2.84553100	0.66597000
O	-1.65453200	-0.47890800	-0.53361600
C	0.61083000	-0.96177300	-0.33490600
O	1.92020900	1.43786600	1.70981000
H	1.37845187	-1.37759918	0.28375809
C	0.88214654	-1.23885390	-1.82527961
C	-0.16441552	-1.18840760	-2.74599192
C	2.17502007	-1.53982919	-2.25458346
C	0.08164861	-1.43924027	-4.09617963
H	-1.18339166	-0.95086783	-2.40775335
C	2.42101537	-1.79107899	-3.60436006
H	3.00006506	-1.57912927	-1.52847706
C	1.37389326	-1.74110665	-4.52536744
H	-0.74379371	-1.40014805	-4.82168536
H	3.43983124	-2.02905299	-3.94300286
H	1.56780823	-1.93944408	-5.58948950

3. References

1. Andrés, J.; Safont, V.S.; Oliva, M.; Caster, K.L.; Goulay, F. A bonding evolution theory study of the reaction between methylidyne radical, CH(X2Π), and cyclopentadiene, C5H6. *Int. J. Quantum. Chem.* **2022**, *122(11)*, e26892.
2. Berski, S.; Andrés, J.; Silvi, B.; Domingo, L.R. The Joint Use of Catastrophe Theory and Electron Localization Function to Characterize Molecular Mechanisms. A Density Functional Study of the Diels−Alder Reaction between Ethylene and 1,3-Butadiene. *J. Phys. Chem. A*, **2003**, *107*, 6014-6024.
3. Berski, S.; Andrés, J.; Silvi, B.; Domingo, L.R. New findings on the Diels− Alder reactions. An analysis based on the bonding evolution theory. *J. Phys. Chem. A*, **2006**, *110*, 13939-13947.
4. Polo, V.; Andrés, J.; Berski, S.; Domingo, L.R.; Silvi, B. Understanding reaction mechanisms in organic chemistry from catastrophe theory applied to the electron localization function topology. *J. Phys. Chem. A*, **2008**, *112*, 7128-7137.
5. Andrés, J.; González-Navarrete, P.; Safont, V.S. Unraveling reaction mechanisms by means of quantum chemical topology analysis. *Int. J. Quant. Chem.*, **2014**, *114*, 1239-1252.
6. Andrés, J.; Berski, S.; Domingo, L.R.; Polo, V.; Silvi, B. Describing the molecular mechanism of organic reactions by using topological analysis of electronic localization function. *Curr. Org. Chem*. **2011**, *15*, 3566-3575.

7. Andrés, J.; Gracia, L.; González-Navarrete, P.; Safont, V.S. Chemical structure and reactivity by means of quantum chemical topology analysis. *Comp. Theor. Chem*., **2015**, *1053*, 17-30.
8. Domingo, L.R. Molecular electron density theory: a modern view of reactivity in organic chemistry. *Molecules*, **2016**, *21*, 1319.
9. Becke, A.D.; Edgecombe, K.E. A simple measure of electron localization in atomic and molecular systems. *J. Chem. Phys*., **1990**, *92*, 5397.
10. Silvi, B. The synaptic order: a key concept to understand multicenter bonding. *J. Mol. Struct.,* **2002**, *614*, 3-10.
11. Fukui, K. Formulation of the reaction coordinate. *J. Phys. Chem*., **1970**, *74*, 4161.
12. Bader, R.F.E. *Atoms in Molecules. A Quantum Theory*, Claredon Press, Oxford, U.K, **1990**.
13. Abraham, R.H.; Shaw, C.D. *Dynamics: The Geometry of Behavior*, Addison-Wesley, Redwood City, CA, **1992**.
14. Parr, R.G.; Yang, W. *Density Functional Theory of Atoms and Molecules*, Oxford University Press, New York, **1989**.
15. Silvi, B.; Savin, A. Classification of chemical bonds based on topological analysis of electron localization functions. *Nature*, **1994**, *371*, 683-686.
16. Savin, A.; Silvi, B.; Colonna, F. Topological analysis of the electron localization function applied to delocalized bonds. *Can. J. Chem*., **1996**, *74*, 1088-1096.
17. Savin, A.; Becke, A.D.; Flad, J.; Nesper, R.; Preuss, H.; Vonschnering, H.G.A. A new look at electron localization. *Angew. Chem. Int. Ed*., **1991**, *30*, 409-412.
18. Savin, A.; Nesper, R.; Wengert, S.; Fassler, T.F. ELF: The electron localization function. *Angew. Chem. Int. Ed*., **1997**, *36*, 1808-1832.
19. Savin, A. On the significance of ELF basins. *J. Chem. Sci*., **2005**, *117*, 473-475.
20. Thom, R. Structural Stability and Morphogenesis: An Outline of a General Theory of Models, Inc., Reading, Mass, London-Amsterdam, **1976**.
21. Woodcock, A.E.R.; Poston, T. A Geometrical Study of Elementary Catastrophes, Spinger-Verlag, Berlin, **1974**.
22. Gilmore, R. Catastrophe Theory for Scientists and Engineers, Dover, New York, **1981**.
23. Kącka-Zych, A.; Jasiński, R. A DFT study on the molecular mechanism of the conjugated nitroalkenes polymerization process initiated by selected unsaturated nucleophiles. *Theor. Chem. Acc*., **2020**, *139*, 119.
24. Kącka-Zych, A. Push-pull nitronates in the [3+ 2] cycloaddition with nitroethylene: Molecular Electron Density Theory study. *J. Mol. Graph. Model*. **2020**, *97*, 107549.
25. Kącka-Zych, A. Understanding the molecular mechanism of the rearrangement of internal nitronic ester into nitronorbornene in light of the MEDT study. *Molecules*, **2019**, *24*, 462.
26. Chamorro, E.; Fuentealba, P.; Savin, A. Electron probability distribution in AIM and ELF basins. *J. Comput. Chem.*, **2003**, *24*, 496-504.
27. Chamorro, E. The nature of bonding in pericyclic and pseudopericyclic transition states: Thermal chelotropic decarbonylations. *J. Chem. Phys*., **2003**, *118*, 8687.

28. Chamorro, E.; Notario, R.; Santos, J.C.; Pérez, P. A theoretical scale for pericyclic and pseudopericyclic reactions. *Chem. Phys. Lett.*, **2007**, *443*, 136-140.
29. Domingo, L.R.; Chamorro, E.; Pérez, P. Understanding the reactivity of captodative ethylenes in polar cycloaddition reactions. A theoretical study. *J. Org. Chem.*, **2008**, *73*, 4615-4624.
30. Domingo, L.R.; Chamorro, E.; Pérez, P. Understanding the mechanism of non-polar Diels–Alder reactions. A comparative ELF analysis of concerted and stepwise diradical mechanisms. *Org. Biomol. Chem.*, **2010**, *8*, 5495-5504.
31. Berski, S.; Ciunik, Z. The mechanism of the formation of the hemiaminal and Schiff base from the benzaldehyde and triazole studied by means of the topological analysis of electron localisation function and catastrophe theory. *Mol. Phys*., **2015**, *113*, 765-781.
32. Ríos-Gutiérrez, M.; Pérez, P.; Domingo, L.R. A bonding evolution theory study of the mechanism of [3+ 2] cycloaddition reactions of nitrones with electron-deficient ethylenes. *RSC Adv.*, **2015**, *5*, 58464-58477.
33. Domingo, L.R.; Ríos-Gutiérrez, M.; Pérez, P. Unveiling the Lewis acid catalyzed Diels–Alder reactions through the molecular electron density theory. *Molecules*, **2020**, *25*, 2535.
34. Domingo, L.R.; Ríos-Gutiérrez, M.; Pérez, P. A molecular electron density theory study of the participation of tetrazines in aza-Diels–Alder reactions. *RSC Adv*., **2020**, *10*, 15394-15405.
35. Domingo, L.R.; Ríos-Gutiérrez, M.; Pérez, P. A molecular electron density theory study of the enhanced reactivity of aza aromatic compounds participating in Diels–Alder reactions. *Org. Biomol. Chem*., **2020**, *18*, 292-304.
36. Frisch, M. J.; Trucks, G. W.; Schlegel, H. B.; Scuseria, G. E.; Robb, M. A.; Cheeseman, J. R.; Montgomery, J. A.; Vreven, T. J.; Kudin, K. N.; Burant, J. C.; Millam, J. M.; Iyengar, S. S.; Tomasi, J.; Barone, V.; Mennucci, B.; Cossi, M.; Scalmani, G.; Rega, N.; Petersson, G. A.; Nakatsuji, H.; Hada, M.; Ehara, M.; Toyota, K.; Fukuda, R.; Hasegawa, J.; Ishida, M.; Nakajima, Y.; Honda, O.; Kitao, O.; Nakai, H.; Klene, M.; Li, X.; Knox, J. E.; Hratchian, H. P.; Cross, J. B.; Adamo, C.; Jaramillo, J.; Gomperts, R.; Stratmann, R. E.; Yazyev, O.; Austin, A. J.; Cammi, R.; Pomelli, C.; Ochterski, J. W.; Ayala, P. Y.; Morokuma, K.; Voth, G. A.; Salvador, P.; Dannenberg, J. J.; Zakrzewski, V. G.; Dapprich, S.; Daniels, A. D.; Strain, M. C.; Farkas, M. C.; Malick, D. K.; Rabuck, A. D.; Raghavachari, K.; Foresman, J. B.; Ortiz, J. V.; Cui, Q.; Baboul, A. G.; Clifford, S.; Cioslowski, J.; Stefanov, B. B.; Liu, G.; Liashenko, A.; Piskorz, P.; Komaromi, I.; Martin, R. L.; Fox, D. J.; Keith, T.; Al-Laham, M. A.; Peng, C. Y.; Nanayakkara, A.; Challacombe, M.; Gill, P. M. W.; Johnson, B.; Chen, W.; Wong, M. W.; Gonzalez, C.; Pople, J. A. Gaussian 16 rev A.1 Gaussian Inc, Wallingford CT, **2016**.
37. Cossi, M.; Rega, N.; Scalmani, G.; Barone, V. Energies, structures, and electronic properties of molecules in solution with the C PCM solvation model. *J. Comput. Chem.* **2003**, *24(6)*, 669–681.

38. Geerlings, P.; De Proft, F.; Langenaeker, W. Conceptual density functional theory. *Chem. Rev.* **2003**, *103(5)*, 1793–1874.
39. Domingo, L.R. Advances in conceptual DFT: electrophilicity, nucleophilicity, and beyond. *RSC Adv.* **2014**, *4(60)*, 32415–32428.
40. Domingo, L.R.; Aurell, M.J.; Pérez, P.; Contreras, R. Quantitative characterization of the global electrophilicity power of common diene/dienophile pairs in Diels–Alder reactions. *Tetrahedron*. **2002**, *58(20)*, 4417–4423.
41. Parr, R.G.; Szentpály, L.; Liu, S. Electrophilicity index. *J. Am. Chem. Soc.* **1999**, *121(9)*, 1922–1924.
42. Domingo, L.R.; Pérez, P. The nucleophilicity N index in organic chemistry. *Org. Biomol. Chem*. **2011,** *9(36)*, 7168–7175..
43. Domingo, L.R.; Pérez, P.; Sáez, J.A. Understanding the local reactivity in polar organic reactions through electrophilic and nucleophilic Parr functions. *RSC Adv.* **2013**, *3(4*), 1486–1494.
44. Noury, S.; Krokidis, X.; Fuster, F.; Silvi, B. Computational tools for the electron localization function topological analysis. *Comput Chem.* **1999**, *23(6)*, 597–604.
45. Dennington, R.; Keith, T.A.; Millam, J.M. GaussView, Version 6.1. Shawnee Mission, KS: Semichem Inc.; **2016**.
46. Ahrens, J.; Geveci, B.; Law, C. ParaView: an end user tool for large data visualization. In: Visualization Handbook. Elsevier; **2005**.
47. Ayachit, U. The ParaView Guide: A Parallel Visualization Application. Kitware; **2015**.
